# Intraperitoneal transplant of Hepatocytes co-Encapsulated with mesenchymal stromal cells in modified alginate microbeads for the treatment of acute Liver failure in Pediatric patients (HELP)—An open-label, single-arm Simon’s two stage phase 1 study protocol

**DOI:** 10.1371/journal.pone.0288185

**Published:** 2023-07-25

**Authors:** Emer Fitzpatrick, Celine Filippi, Barath Jagadisan, Dharshene Shivapatham, Hanish Anand, Mike Lyne, Katherine-Daisy Stroud, Rebecca Newton, Marc DeLord, Abdel Douiri, Anil Dhawan

**Affiliations:** 1 Pediatric Liver GI and Nutrition Centre and Mowat Labs, King’s College Hospital, London, United Kingdom; 2 King’s College London, London, United Kingdom; 3 Biomedical Research Centre, Guy’s and St Thomas’ NHS Foundation Trust and King’s College London, London, United Kingdom; 4 King’s Health Partners Clinical Trials Office, London, United Kingdom; 5 School of Population Health and Environmental Sciences, King’s College London, London, United Kingdom; PLOS: Public Library of Science, UNITED KINGDOM

## Abstract

**Background:**

Pediatric acute liver failure (PALF) carries a high mortality without liver transplantation (LT) in children. Liver transplantation, though lifesaving, is limited by timely donor organ availability, the risks of major surgery and complications of life-long immunosuppression. Hepatocyte transplantation (HT) improves synthetic and detoxification functions in small animal models. The encapsulation of hepatocytes in alginate protects it from the recipient immune system while the intraperitoneal route of administration allows large volumes to be infused. The safety and possibly short-term efficacy of encapsulated hepatocytes has been observed in a named patient use. A novel type of microbeads (HMB002) has been developed, using a modified alginate and mesenchymal stromal cells (MSCs). Its safety and medium-term efficacy need to be studied in the context of clinical study while optimizing the hepatocyte function and viability using modifications of the alginate and MSCs co-encapsulation.

**Methods:**

A single centre, non-randomised, open-label, single-arm Simon’s two stage study will be conducted to evaluate the safety, biological activity and tolerability of transplantation of a single intraperitoneal dose of microbeads made from an optimum combination of a modified alginate, MSCs and hepatocytes in 17 patients less than 16 years of age with acute liver failure (Stage 1: 9 patients and Stage 2: 8 patient). Safety will be assessed by documenting moderate to severe (including life threatening and death) adverse events due to HMB002 in the first 52 weeks post-procedure. Tolerability will be assessed by observing the proportion of initiated infusions where >80% of infusion is received by the patient. Biological activity will be reflected in patient survival with native liver at 24 weeks post treatment.

**Discussion:**

HMB002, if safe and efficacious in acute liver failure, could be a bridge until the liver regenerates or a suitable organ becomes available. There are multiple advantages to using HT. HT, when delivered by the intraperitoneal route, is less invasive than LT. Hepatocytes from a single donor liver can be used to treat multiple patients. Cryopreserved cells provide an off-the-shelf emergency treatment in PALF. When encapsulated, alginate encapsulation of hepatocytes precludes the need for immunosuppression unlike in LT.

## Introduction

Pediatric acute Liver Failure (PALF) carries a high mortality without liver transplantation (LT) in children [1]. Liver transplantation, though lifesaving, is limited by timely donor organ availability, the risks of major surgery and complications of life-long immunosuppression. Given the regenerative potential of the liver, complete regeneration of the failing liver can be achieved after PALF. This has been demonstrated in auxiliary liver transplantation for PALF where 70% of recipients were able to regenerate their native liver [2]. Hepatocyte transplantation (HT) improves synthetic and detoxification functions in small animal models of acute liver failure [3]. HT, if shown to be safe and efficacious in acute liver failure, could bridge the patient to full recovery if the native liver regenerates or, failing this, support them until a suitable organ is available for LT. HT has multiple advantages over LT. Hepatocyte isolation can yield billions of cells from a single donor liver, that can be used to treat multiple patients. These cells can be cryopreserved for a number of years, providing an off-the-shelf treatment in emergency situations such as PALF. The encapsulation of hepatocytes in alginate protects them from the recipient immune system and precludes the need for immunosuppression, unlike in LT. The intraperitoneal route of administration of microbeads allows large volumes to be infused. HT when delivered intraperitoneally is less invasive than LT. The safety and possibly short-term efficacy of encapsulated hepatocytes has been observed in a named patient use [4]. Mesenchymal stromal cells are known to improve the viability and function of hepatocytes [5, 6]. We have now developed a novel microbead prototype (HMB002) using modified alginate and mesenchymal stromal cells (MSCs) under good manufacturing practice (GMP) standards. Safety and medium-term efficacy (up to 24 weeks while awaiting liver regeneration) need to be studied.

## Materials and methods

### Trial objectives

The trial objective is to evaluate if HMB002 is a safe and effective form of liver support in Pediatric acute liver failure to bridge the patient to native liver survival or liver transplantation. This study will involve intraperitoneal infusion of HMB002. The exact formulation of HMB002 is proprietary but the infusate will consist of alginate beads suspended in transplant medium (1:1 v/v). This is given as a single infusion under ultrasound guidance while observing for any adverse events related to increase in intrabdominal pressure. This will be in addition to standard of care in Pediatric acute liver failure. The beads will be removed using laparoscopy prior to discharge of the patient from hospital, or at time of transplantation, to minimize or eliminate the risk of adhesions within the peritoneal cavity.

#### Primary objective

To evaluate the safety, biological activity and tolerability of transplantation of a single dose of microbeads made from optimum combination of modified alginate, MSCs and hepatocytes to Pediatric patients with acute liver failure.

#### Secondary objective

To establish proof of concept of the transplantation of microbeads made from the optimum combination of modified alginate, MSCs and hepatocytes. To inform the sample size and confidence intervals to design a larger randomized clinical trial.

### Trial registration

EudraCT Number: 2019-000316-29, ClinicalTrials.gov Identifier: NCT05491135.

### Protocol version

Version 3.2 (15th Dec 2022).

### Trial design

This is an, open-label, single-arm, single centre study. A Simon’s two-stage design for a one-sample exact test will be used. We assume a one-year survival rate of 0.20 or less under the null hypothesis, and 0.5 or more under the alternative, with 80% power and 5% type I error rate.

The primary outcome survival measure is set at 24-weeks. Although response to treatment is anticipated to be within the first 4–6 weeks, short to medium term morbidity in children who have been critically unwell in the Pediatric Intensive Care Unit (PICU), Neonatal Intensive Care Unit (NICU) or High Dependency Unit (HDU), is best determined at a longer time point such as 24 weeks. This way almost all possible later deaths due to the acute liver failure (or early post-transplant deaths) will be effectively captured.

#### Sample size

Simon’s two-stage design for a one-sample exact test was estimated, assuming a one-year survival rate of 0.20 or less under the null hypothesis, and 0.5 or more under the alternative, with 80% power and 5% type I error rate. A total of 17 patients will be recruited into the study (at the end of stages 1 and 2). 9 patients will be recruited into the study in the first stage. Once 9 patients have completed their 24 weeks visit, the study will stop for futility if only 2 or fewer patients have survived with the native liver. At the end of the second stage, if 7 or more patients out of the 17 enrolled have survived with native liver at 24 weeks post HMB002 treatment, this will demonstrate proof of concept. DSMB approval is required for progression to Stage 2, where the trial will continue to recruit a further 8 patients. this will be extended to 17 patients as part of the Simon two stage design if there is evidence to support continuation. A long-term follow-up period will be used to monitor safety over a 10-year period post investigational medicinal product (IMP) infusion.

#### Trial duration and flowchart

**Fig 1:**
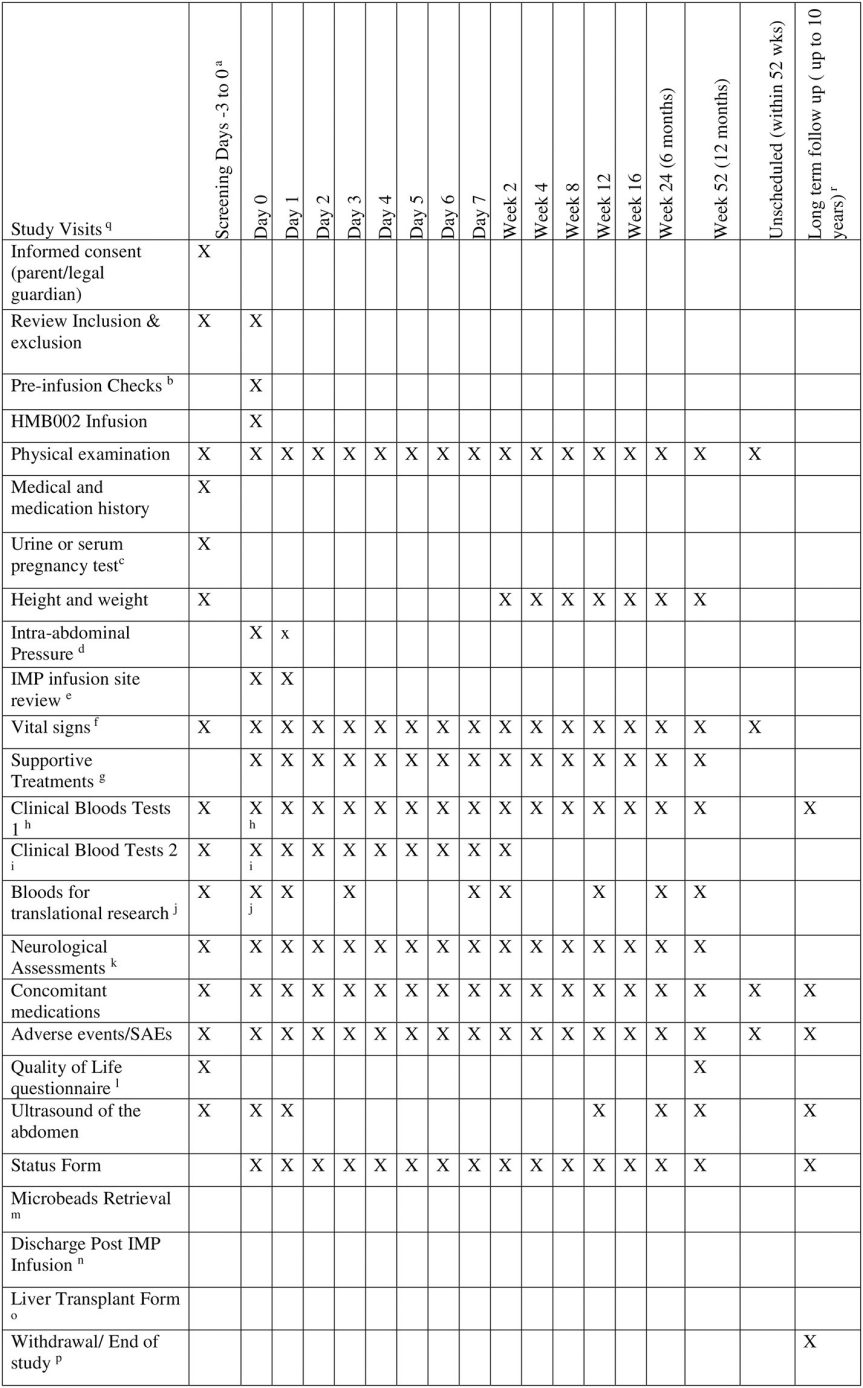
Schedule of assessment. ^a^ Screening and infusion may take place on the same day (Day 0) depending on clinical decision by delegated physician. ^b^ HMB002 infusion will be performed on Day 0 following complete review of all inclusion and exclusion criteria and completion of all pre-infusion checks. See section 4.3 Dosing regimen for details on dosing intervals between subsequent patients. ^c^ Urine or serum pregnancy test (in cases of oliguria) will be performed in females of child bearing potential (FOCBP). FOCBP will only be included after a confirmed negative pregnancy test at screening. Pregnancy is an absolute contraindication to inclusion into the study. However, patients will be hospitalised at the time of screening and until recovery of their native liver or after transplant. Hence, during hospitalisation it is assumed that patients will be sexually abstinent, which is considered a highly effective method of contraception. Therefore, females of child bearing age are eligible for the trial without need for any additional testing or contraception. Should the patient survive with or without liver transplantation, participants will be advised as to highly effective methods of contraception if this is this is appropriate. ^d^ Intra-abdominal pressure will be measured using urinary catheter where possible, at pre-dose (10 minutes prior to start of IMP infusion) and post dose: 1 hr, 8 hrs and 24 hrs (day1). ^e^ IMP Infusion site to be reviewed at 2 hrs, 4hrs, 6 hrs, 8hrs, 12 hrs and 24 hrs post infusion. ^f^ Vital signs include Blood Pressure (BP), Pulse Rate (PR), Respiratory Rate (RR), Temperature and Oxygen Saturation (02 Sats). On Day 0, vital signs will be taken at Pre-dose (approximately 30 mins and 10 mins prior to start of IMP infusion) and Post-dose: 30mins, 1hr, 1.5hrs, 2 hrs, 2.5 hours, 3 hrs, 3.5 hrs 4 hrs, 8 hrs, 12 hrs and 24 hrs (Day1). ^g^ Recording of ventilator settings, ionotropic support (including drugs and dose) and need for renal replacement therapy will be collected as part of standard supportive treatment. On Day 0, supportive treatment data will be collected at pre-dose (30 mins and 10 mins prior to start of IMP infusion and post dose: 30mins, 1hr, 1.5hr, 2hrs, 2.5hrs, 3hrs, 3.5hrs, 4hrs, 8hrs, 12 hrs & 24 hrs(Day 1). ^h^ Clinical Blood Tests 1 include haematology (full blood count and differentials), clotting factors (INR, APTT, fibrinogen) liver function tests (ALT, AST, Creatine Kinase, Total bilirubin, Conjugated bilirubin (only done at screening and if clinically applicable), ALP, Albumin, total protein), urea and electrolytes (sodium, potassium, chloride, urea, creatinine) and Ammonia. At Day 0, bloods will be taken pre-dose (between -4 to -1 hour prior to IMP infusion) and post dose: 1 hr, 8hrs, 16hrs and 24hrs (day1). ^i^ Clinical Blood tests 2 include blood glucose, lactate and blood gases (pH, Partial pressure of O2, Partial pressure of carbon dioxide, standard bicarbonate). On Day 0, these blood will be taken pre dose (10mins prior to start of IMP infusion) and post dose: 1hr, 2hrs, 4hrs, 8hrs, 16hrs, 24hrs (day1). ^j^ Bloods for translational research on Day 0 –to be taken approximately 10 mins prior to IMP infusion and 1 hour post IMP infusion. Aliquots of certain human products (fresh frozen plasma, cryoprecipitate and albumin) administered as part of standard of care will also be sent as controls. ^k^ Neurological Assessment includes Glasgow Coma Scale and Pupil response. ^l^ PedsQL^™^ Quality of Life Inventory questionnaires for parent and child will be optional; completed at screening and week 52 visits. ^m^ The beads will be removed using laparoscopy prior to discharge of the patient from hospital or washed out at the time of transplantation, whichever occurs first. Usually this will be within 4 weeks of the procedure but in any case, microbeads will be retrieved within 24 weeks of HMB002 infusion. ^n^ Live**r** Transplant form will be completed for those patients who go onto receive a transplant within the duration of the study. ^o^ Patients will be discharged from hospital following recovery with either native liver or post liver transplant. Discharge post IMP infusion form to be completed. ^p^ End of study/withdrawal form will be completed following completion of the study or for those patients who did not complete the study. ^q^ All outpatient study visits following discharge, will have a flexible window of +/- 3 days except week 52 visits where +/- 7 days is permitted. ^r^ Long term follow up data will be collected annually within +/- 1 month window. FBC, LFT and abdominal ultrasound will be collected annually until year 5 post IMP. SAEs and associated concomitant medications (except SAEs excluded from reporting) will be collected until end of study.

**Fig 2:**
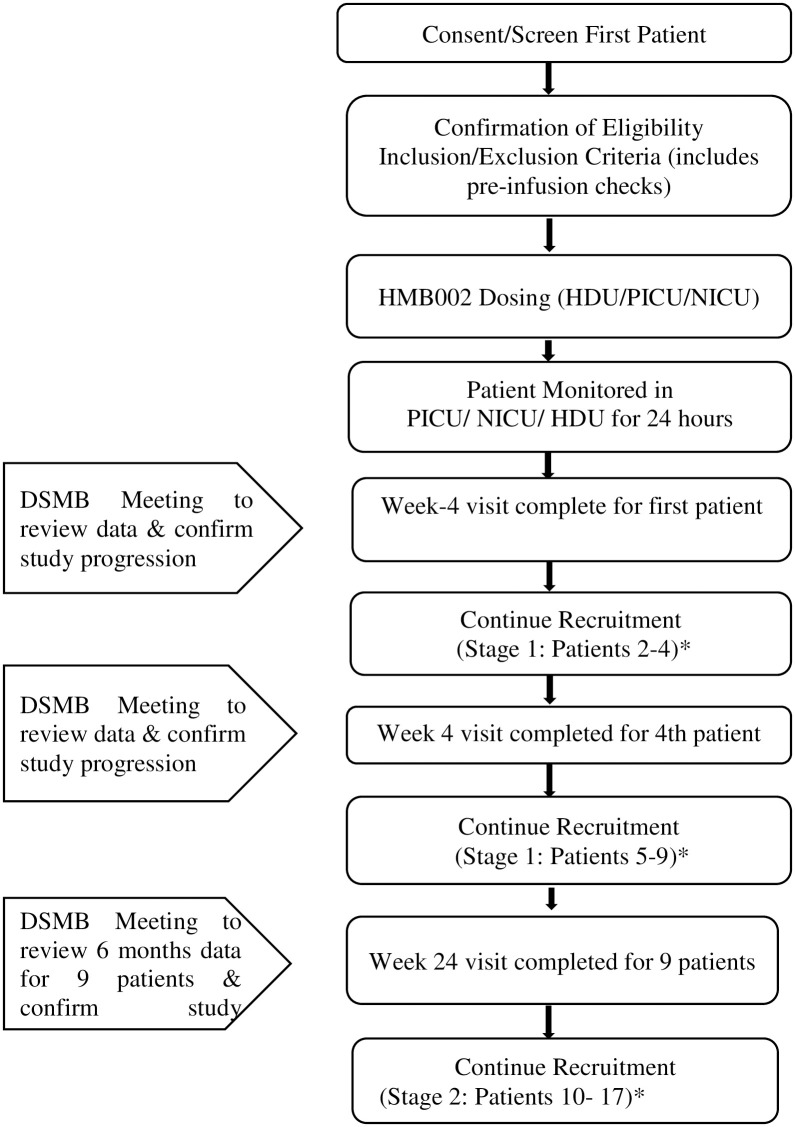
Trial flowchart. *Interim DSMB meetings as advised by DSMB Chair for the duration of the study. # Study will be terminated if 2 or fewer patients survive with native liver at 24 weeks post HMB002 infusion, after stage 1 of the trial. A total of 17 patients will be recruited into the study (stage 1 = 9 patients and stage 2 = 8 patients).

We will aim to recruit the 17 patients (stage 1 and 2) in approximately 36 months. Each individual patient will be intensively followed up for 52 weeks after receiving HMB002 infusion and then for long-term safety monitoring for a further 9 years. The end of the trial will be defined as the last patient 10-year annual follow up post IMP infusion. The duration of the study will also depend on the progression of the study from stage 1 to stage 2. The schedule of assessment has been explained in Fig 1. The trial flow chart is depicted in Fig 2.

#### Study setting

Patients will be recruited from a single academic hospital, the Pediatric Liver Centre King’s College Hospital, London. This is one of 3 centres in the UK to which children with acute liver failure are referred. All study visits up to 52 weeks will be conducted at King’s College Hospital (KCH).

#### Eligibility criteria

*Inclusion criteria*.

I. Infant or child (male or female) under the age of 16 years at recruitment.
II. Written informed consent obtained from a parent / legal guardian;
III. Presence of ALF defined as a multisystemic disorder in which severe impairment of liver function with or without encephalopathy^a^ occurs in association with hepatocellular necrosis reflected as synthetic liver failure in a child with no recognised underlying chronic liver disease. Children must fit one of the ALF categories as described in Appendix 1^b^ in S2 File;
IV. Willing and able to comply with the study visit schedule.

a—Diagnosis of encephalopathy may not be possible in infants and small children

Other parameters become more relevant

b—Children who meet inclusion criteria as above but would otherwise not be suitable for liver transplant because of progressive neurological disease for example, will not be excluded from the trial unless they also have exclusions as detailed in criteria for the trial.

*Exclusion criteria*.

I. Severe ascites causing high intra-abdominal pressure and / or respiratory compromise;
II. Intra-abdominal sepsis suspected or proven;
III. Clinical condition too unstable to tolerate procedure without compromise;
IV. Proven pre-existing allergy or intolerance to alginate on medical history;
V. Proven pre-existing allergy to gentamicin on medical history;
VI. Intraperitoneal or intra-abdominal malignancy;
VII. Adhesions or fistulae to anterior abdominal wall;
VIII. Children who weigh in excess of 33kg
IX. Pregnant or lactating patients (positive pregnancy test for females of child bearing potential at screening).
X. Female patients of childbearing potential who are not willing to use highly effective methods of contraception to prevent pregnancy or abstain from heterosexual activity for 52 weeks post treatment.
XI. Male patients who are not willing to use an effective method of contraception (condom, vasectomy, sexual abstinence) for 52 weeks post study treatment, when engaging in sexual activity with a female of childbearing potential;
XII. Participation in concurrent therapeutic trial for PALF;
XIII. Imminent liver transplantation expected within 12 hours of infusion;
XIV. Total hepatectomy;
XV. Dependent on extracorporeal membrane oxygenation;
XVI. Previous liver transplant

#### Replacement of subjects

Eligible patients who do not receive IMP infusion following screening, or who do not tolerate the procedure will be replaced for the purpose of maintaining trial numbers.

#### Ethics approval and consent to participate

Research ethic committee approval was obtained (approval number: 22/LO/0292). Written, informed consent to participate will be obtained from all participants. The Chief Investigator (CI), or person to whom the CI delegates the responsibility will obtain written informed consent from each parent/legal guardian. All trial investigators seeking consent must also receive Human Tissue Act training for the taking of consent involving tissues and cells used as part of the trial and be up-to-date with their Good Clinical Practice training. Model consent form and other related documentation given to participants and authorized surrogates will be provided on request. Additional 1ml of blood will be taken for research assays at the same time as clinical blood tests. Consent for research blood samples will be optional.

#### Intervention

*HMB002 production*. The final investigational product will be made of:

1. primary human hepatocytes isolated from donor organs, procured through NHSBT after specific consent for liver cell isolation, in the NIHR/Welcome Trust Cell Therapy Unit at King’s College Hospital (Licence Number 11062)
2. MSCs procured after consent and isolated in King’s College Hospital GMP Cell Therapy Unit above.
3. a modified alginate produced under GMP regulations.
4. transplant medium (CMRL-1066 with Human Albumin Solution).

The final product is named as HMB002. The alginate microbeads will be manufactured in a GMP unit, from cells isolated/prepared and cryopreserved following EU-GMP regulation. The final product is packaged as 20mL/kg final volume in sterile syringes for infusion, once quality control checks are satisfactory. Children who weigh in excess of 33kg cannot be treated within the study due to current capacity for HMB002 manufacture. Children with a known allergy to gentamicin will also be excluded from the trial as the MSCs are manufactured using gentamicin. The donation, procurement and testing of the human tissues and cells are in conformity with the relevant regulations. The donation of the human cells used to manufacture the IMP is not considered a part of this trial.

*IMP packaging and storage*. The final product (HMB002) is packed in sterile syringes and kept at 2–8°C from time of manufacture to administration. The IMP will be administered shortly after production and always within 8 hours from time of manufacture. Release criteria for the final product include checks of sterility, bead characteristics, endotoxinemia and mycoplasma testing. The treating physician will be informed if the product fails any of the final release tests.

*Dosing regimen*. The route of administration of the product is intra-peritoneal with a 14-16G cannula under ultrasound guidance by a physician. A single dose (weight-dependent) will be used in all patients. There are no increments of dosage. The dose will be administered as soon as possible following parental/legal guardian consent and screening of the patient, as these patients are critically ill. The infusion will be administered at a rate of approximately 150-200ml per hour under antibiotic prophylaxis and close monitoring. This dose has been determined using in vitro, in vivo and previous clinical pilot experience. An infusion of more than 80% of the final product for the patient will be considered as a completed infusion.

#### Criteria for discontinuing or modifying interventions

Where possible intra-abdominal pressure will be measured pre-infusion using urinary catheter. Pre-infusion Checks form will be completed prior to dosing with HMB002.

Pre-infusion checks include:

i. Correction of coagulopathy
ii. Haemoglobin > 7g/dl
iii. Patient has not got tense ascites
iv. Patient is sufficiently stable to tolerate procedure
v. Patient diagnosis / status has not changed from time of screening and consent
vi. No organ transplant is available.
vii. Full PICU / NICU / HDU monitoring in place and staffing sufficient to provide the same monitoring following the infusion.

Whenever the pre-infusion checks reveal that the patient is not suitable for infusion, the procedure is delayed or abandoned. The patient may be scheduled for infusion on a different day if the patient is still eligible and does not have any contraindications for infusion.

#### Outcomes

*Primary endpoints*.

- Safety: Moderate to severe (including life threatening and death) adverse event occurrences due to product in 1^st^ 52 weeks post procedure
- Tolerability: assessed by the proportion of initiated infusions where >80% of the infusion is received by the patient.
- Biological activity: Survival with native liver at 24 weeks post treatment.

*Secondary endpoints*.

- Change in blood marker levels including haematological, biochemical and coagulation
- baseline to 52 weeks post treatment.
- Quality of life measures
- Patient survival with native liver at 52 weeks post treatment
- Patient survival with transplanted or native liver at 24- and 52-weeks post treatment.

*Exploratory end points*

- To assess the assay ‘Hepamorph’ which distinguishes the recipient liver cell derived proteins from the donor liver cell derived proteins using a targeted mass spectrometry approach.
- To analyse viability and function of microbeads which are retrieved from the intraperitoneal cavity either at laparoscopy or transplant for phenotype ex vivo.

#### Participant timeline

**Fig 3:**
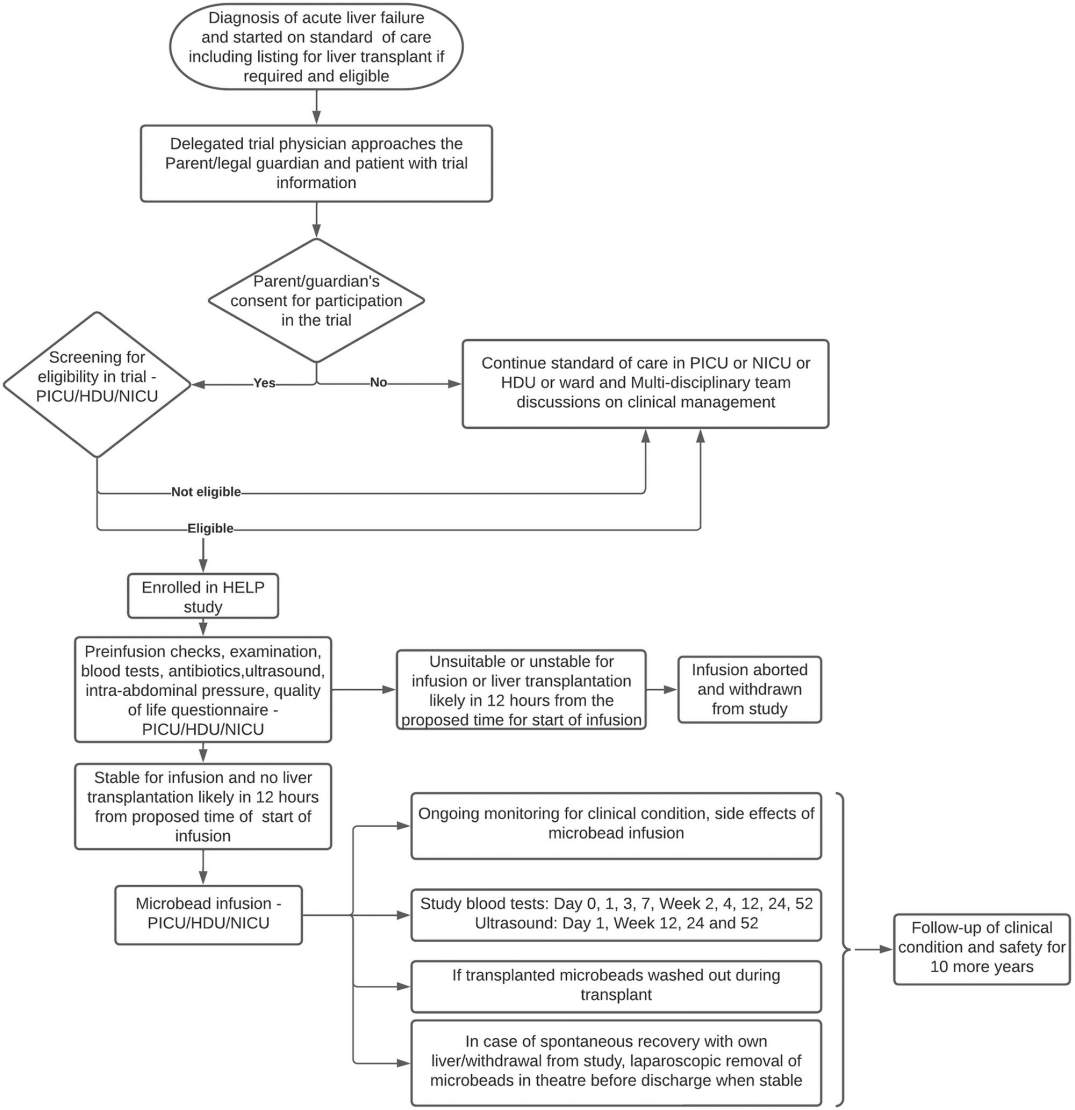
Participant timeline flowchart.

The participant timeline is detailed in Fig 3. The schedule of assessment has been explained in Fig 1.

Safety monitoring will be until 10 years after HMB002 infusion. This will be aligned with routine care. Routine follow up will be conducted annually as a minimum but may be more frequent should the clinical condition of the child or young person require this. For example, if the child has undergone liver transplantation, follow up will be frequent and lifelong.

#### Adverse event reporting and harms

*Specification*, *timing*, *definitions and recording of safety parameters*. *Definitions*. The Medicines for Human Use (Clinical Trials) Regulations 2004 and Amended Regulations 2006 defines Adverse Event (AE), Adverse Reaction (AR), Unexpected Adverse Reaction (UAR), Serious adverse Event (SAE), Serious Adverse Reaction (SAR) and Suspected Unexpected Serious Adverse Reaction (SUSAR).

Documentation of the AEs in the eCRF will be according to the following criteria:

- Description of the AE: diagnosis if known, with signs and symptoms, giving details appropriate to the event,
- Dates of onset and resolution of the AE,
- Severity, grading–using the NCI Common Terminology Criteria for Adverse Events (CTCAE, version 4.0) and detailed below. If not included in CTCAE, this will be a clinical decision and graded as detailed below in severity of adverse events section.
  - Assessment of causal relationship to study treatment (category—definitely, probably, possibly, unlikely, unrelated)
  - Action taken regarding study treatment: none / infusion discontinued / infusion delayed
  - Outcome: complete recovery/not yet recovered/recovered with sequelae/death/unknown. The investigator may be asked to provide follow-up information and/or discharge summaries as needed.

The assignment of causality should be made by the investigator responsible for the care of the participant and discussed with the Chief Investigator (CI) in cases where causality is doubtful.

Safety parameters and adverse event (AE, SAE, SAR and SUSAR) information will be collected from the point of parent/legal guardian consent until week 52 post IMP administration. During the infusion and for at least 24 hours afterwards the patient will be continuously monitored in PICU / NICU / HDU. Intra-abdominal pressure will be monitored via the bladder if possible. Safety will be assessed using history of symptoms which have occurred or worsened since study commencement, physical examination, blood tests including white cell count, haemoglobin, platelets, Urea and electrolytes, liver function tests, lactate, venous blood gas. While an inpatient, need for intervention and escalation of care will also be recorded. Ultrasound may be used to assess for intra-abdominal bleeding.

During long term follow up (post week 52 visit), AEs and SAES will be documented in the medical notes as per routine care practise and reviewed by clinicians. However, only SAEs, SARs and SUSARS will be reported (excluding known complications following liver transplant). Any serious adverse events that are deemed related to the IMP will be considered unexpected (SUSARs). All liver transplants will be reported as an SAE. However, known complications which are universal in patients post liver transplant will not be reported as SAEs to the sponsor, unless they result in death, are considered to be related to the study drug or worse than what would normally be expected.

#### Relevant concomitant care permitted or prohibited during the trial

There are no special dietary or other requirements from the participants. They will have standard of care treatment and monitoring involving adherence to standard treatment regime and receive immunosuppressive treatment in the case of liver transplantation. Data will be collected relating to concomitant medications at every study visit.

#### Provisions for post-trial care

There will be no additional post-trial care apart from what is clinically indicated. No compensation is planned for those who suffer from complications due to trial participation except for what may arise from clinical negligence. As the sponsor, King’s College Hospital NHS Foundation Trust have clinical negligence cover as part of the NHS Clinical Negligence Scheme for Trusts.

#### Recruitment and trial status

Recruitment is yet to begin and the trial is expected to finish recruitment in 3 years. This trial will be communicated within the Pediatric hepatology and intensive care units which makes healthcare providers aware of the study and to encourage recruitment.

#### Data collection and management

*Plans for assessment and collection of outcomes*. Source documentation for the study includes, but is not limited to informed consent forms, medical records, clinical reports, laboratory reports, hospital correspondence and quality of life questionnaires. The validated PedsQL forms used in the study are PedsQL Parent (13–18 years) version 4.0, PedsQL Infant(1-12months) version 1.0, PedsQL Infant(13-24months) version 1.0, PedsQL Parent (2–4 years) version 4.0, PedsQL 5–7 Years version 4.0, PedsQL Parent (5–7 years) version 4.0, PedsQL 8–12 Years version 4.0, PedsQL Parent (8–12 years) version 4.0 and PedsQL 13–18 Years version 4.0.

*Plans to promote participant retention and complete follow-up*. Participants have the right to withdraw from the study at any time for any reason. Should a patient decide to withdraw from study, efforts will be made to explain the importance of remaining on trial follow up and seek permission to continue to allow routine follow-up data to be used for trial purposes (with parent / legal guardian consent). Patients who undergo liver transplant following IMP administration during the study period will not be withdrawn from the study but will continue to undergo monitoring and data collection.

*Data management*. A web based electronic data capture system will be designed, using the InferMed Macro 4 system. It will be hosted at a dedicated secure server within King’s college London. A full audit trial of data entry and any changes to data will be available. Over the course of the trial, the trial monitor will conduct on-site monitoring. The data entered into an electronic form should be verifiable with original source records kept at study centre. Following completion of monitoring and data cleaning of all week-52 post IMP visit data, the site PI will review all the data for each participant and provide electronic sign-off to verify that all the data are complete and correct. At this point, all data will be formally locked for analysis. Safety monitoring data collected during the long term follow up period (week 52 to 10 years post IMP) will be recorded on a separate database and locked following PI review and sign off.

At the end of the trial, all essential documentation will be archived for a minimum of 30 years in a GCP compliant archive facility. The documents that relate to IMP traceability will be retained for a minimum of 30 years and beyond the expiry date of the product.

*Confidentiality*. Patient data will be pseudo-anonymised. All trial data will be stored in line with the Medicines for Humans Use (Clinical Trials) Amended regulations 2006, the Data protection Act 2018 and GDPR.

*Plans for collection*, *laboratory evaluation and storage of biological specimens for genetic or molecular analysis in this trial/future use*. Plasma samples will be collected for HepaMorph panel and Inflammasome tests (proprietary tests in validation). Any surplus plasma sample will be transferred to King’s College Hospital Pediatric liver biobank for long term storage (HTA License no: 12378), following consent from parent/legal guardian.

#### Statistical methods

*Analysis of primary endpoints*.

- The number of moderate to severe adverse event occurrences due to product in 1^st^ 52 weeks post procedure will be reported.
- The proportion of initiated infusions where >80% of the infusion is received by the patient will be reporter.
- Survival with native liver at 24 weeks post treatment and 95% confidence interval will be reported as one minus the cumulative incidence of death with native liver, liver transplant being considered as competing events.

*Analysis of secondary endpoints*.

- Change in blood marker levels (haematological, biochemical and coagulation) will be reported from baseline to 52 weeks post-treatment.
- Change in quality-of-life measures levels will be reported from baseline to 52 weeks post treatment.
- Survival with native liver at 52 weeks post treatment and 95% confidence interval will be reported as one minus the cumulative incidence of death with native liver, liver transplant being considered as competing events.
- Overall survival and 95% confidence interval at 24 weeks and 52 weeks post treatment will be estimated using the Kaplan-Meier method.

*Other reporting*.

A comprehensive report of the collected examination items will be provided according to the protocol’s schedule of assessment.

An analysis of outcomes will be conducted at stage 1 Go/No Go time point for DSMB safety review, when the 9^th^ patient has completed their 24-week post IMP study follow up.

According to the trial design, a first analysis will be conducted once 9 patients have received the proposed treatment and completed 24 weeks of follow up. If two or fewer survive with the native liver at 24 weeks after treatment then the trial will be stopped at this stage. Once 52-week follow up is completed for all 9 patients, primary and secondary endpoint will be analysed according to the protocol specification. If more than 2 survive with the native liver at 24 weeks after treatment, then recruitment will continue to a total of 17 patients (stage 2). Once 52 week follow up will be completed for all 17 patients having received the treatment, primary and secondary endpoints will be analysed according to the protocol specification.

#### Premature discontinuation of study

The trial may be prematurely discontinued by the Sponsor, Chief Investigator or Regulatory Authority on the basis of new safety information or for other reasons given by the DSMB, regulatory authority or ethics committee concerned. The Sponsor and CI reserve the right to stop the trial at any time, for any justifiable reason.

The clinical trial may be prematurely terminated for the following reasons:

- Serious and/or persistent non-compliance with trial protocol
- Non-compliance with ethical standards, regulatory requirements or GCP compliance
- Findings uncovered during monitoring visits, audits or inspections that compromise patient safety or suitability of the site to act as a trial centre
- Recommendation from DSMB
- Failure to meet recruitment targets

The affected trial participants will also be informed promptly and appropriate follow-up visits will be arranged. No further participant data will be collected.

During the course of the study, any of the following events will trigger a halt in patient recruitment and a safety meeting of the DSMB:

- Death or life-threatening event occurs due to a reaction to the product
- Or 2 or more IMP related SAEs of non-lethal non-life-threatening reactions to HMB002.

The trial will be put on hold pending a safety investigation in either case above. If following an internal safety review the sponsor deems it appropriate to restart the trial, this can be done after approval of a substantial amendment.

#### Methods for additional analyses

There are no subgroups to analyse. Calculation of time to death will consider transplantation as a competing risk.

*Methods in analysis to handle protocol non-adherence and missing data*. This is an early phase trial, so that analysis will be conducted per protocol on patients with available data. Patients with incomplete data will therefore be discarded. Patient accrual will be continued until the required number of patients with complete data is reached.

#### Plans to give access to the full protocol, participant level-data and statistical code

Details of the composition of the modified alginate, the ratio and density of hepatocytes and MSCs in the alginate microbeads are withheld in view of a pending patent application. Anonymised patient level data and statistical code may be published subject to approval from the sponsor.

#### Oversight and monitoring

*Composition of the coordinating centre and trial steering committee*. The Trial Management Group will be formed comprising the CI, other lead investigators, core study team including statisticians, clinical trial manager, chief scientist research, research nurse, GMP team. The trial management team will be responsible for the day-to-day management of the trial activities and will meet on a regular basis to discuss any trial related activities or issues.

*Composition of the data monitoring committee*, *its role and reporting structure*. In view of the need for rapid decision making and a high level of involvement by board members who will need to be experts in the field of Pediatric liver failure and first in man studies, the study will be overseen by a single data and safety monitoring board (DSMB). The DSMB will be constituted prior to study opening, comprising of an Independent Chair, Independent Clinicians (s) and an Independent Statistician. The DSMB will review individual and cumulative data to evaluate safety, study conduct, scientific validity and integrity of the trial. The DSMB will meet prior to initiation of study recruitment to agree on the type and format of data reports and sign the DSMB charter. Further details on the DSMB membership and terms of reference will be provided in the DSMB charter.

Timing of Meetings subject to agreement by members of DSMB are:

- prior to initiation of study recruitment
- Safety data review 4 weeks after IMP infusion of first patient
- Safety data review 4 weeks after IMP infusion of fourth patient
- Safety data review 24 weeks after IMP infusion of 9th patient (End of stage 1—GO/ No GO Decision)
- Safety data review at 52 weeks after IMP infusion of 17th patient
- Safety data review at 2 years after IMP infusion of all patients and subsequent review during the long term follow up period.

DSMB meetings will also be convened if:

- Death or life-threatening event occurs due to a reaction to the product
- 2 or more serious cases of non-lethal non-life-threatening reactions to HMB002.
- Or at any time deemed necessary by the DSMB Chair due to safety concerns arising at any time during the duration of the study.

*Frequency and plans for auditing trial conduct*. The CI will allow the sponsor, designated trial monitors, and when necessary, members of the REC or representatives of the regulatory authorities to review, monitor, audit and/ or inspect the trial by providing direct access to source data and other documents (e.g. patients’ case sheets, blood test reports, histology reports etc.).

#### Plans for communicating important protocol amendments and dissemination of outcomes

The Chief Investigator will submit any further amendments to the protocol and a final report at conclusion of the trial to the sponsor, the REC and the MHRA within the timelines defined in the Regulations. All data and results generated from this trial are confidential. Agreement from the sponsors will be required prior to the disclosure of any trial related data. It is intended that the results of the study will be reported and disseminated at international conferences and in peer-reviewed scientific journals.

## Discussion

Our previous alginate encapsulation technique used PRONOVA^™^ UP, an ultrapure alginate that satisfied GMP standards. We had optimised the alginate percentage, cell density and bead size allowing the best cell survival, and demonstrated that this alginate was not immunogenic using in vitro tests [7]. However, this alginate led to limited in vitro cell survival. The development of HMB002 followed the hypothesis that co-encapsulation of hepatocytes with MSCs, in modified alginate microbeads, would substantially improve the survival and function of hepatocytes and be a feasible and effective form of cellular therapy for acute liver failure [5, 6].

This trial, while demonstrating the safety of HMB002 would support the design of a larger study if the intervention is found to be safe. The trial will also report 52-week survival outcome. This is not anticipated to change from 24-week outcome as this trial is to attempt to rescue acute liver failure. If the 52-week outcome differs from the 24-week outcome this observation will be an important consideration in the design of any future trial. The target population is too small and too unwell to do any significant dose escalation study.

## Supporting information

S1 Checklist SPIRIT 2013 checklist: Recommended items to address in a clinical trial protocol and related documents*. (DOCX)

S1 File (DOCX)

S2 File (PDF)

## Abbreviations

AE: Adverse Event
ALF: Acute liver failure
AR: Adverse Reaction
DSMB: Data safety and monitoring board
GMP: Good manufacturing practice
HDU: High Dependency Unit
HT: Hepatocyte transplantation
IMP: Investigational medicinal product
LT: Liver transplantation
MHRA: Medicines and Healthcare products Regulatory Agency
MSC: Mesenchymal stromal cell
NICU: Neonatal Intensive Care Unit
PALF: Pediatric acute liver failure
PICU: Pediatric Intensive Care Unit
REC: Research Ethics Committee
SAE: Serious adverse Event
SAE: Serious Adverse Reaction (SAR)
SUSAR: Suspected Unexpected Serious Adverse Reaction
UAR: Unexpected Adverse Reaction
