# Supplementary material for: Intraperitoneal transplant of Hepatocytes co-Encapsulated with mesenchymal stromal cells in modified alginate microbeads for the treatment of acute Liver failure in Pediatric patients (HELP)—An open-label, single-arm Simon’s two stage phase 1 study protocol
Source: PLoS One. 2023 Jul 25;18(7):e0288185. doi: 10.1371/journal.pone.0288185 (PMC10368261; doi:10.1371/journal.pone.0288185)
Supplement: S1 File — (DOCX) [file pone.0288185.s002.docx]

**Definitions:**Females of child bearing potential are females who have experienced menarche and are not surgically sterilised (e.g. by tubal occlusion, hysterectomy, bilateral salpingectomy) or post-menopausal (defined as at least 1 year since last regular menstrual period).

Highly effective methods of birth control are those with a failure rate of < 1% per year when employed consistently and correctly.

Highly effective methods of contraception as per HMA / CTFG working group are combined (oestrogen and progestogen containing) hormonal contraception associated with inhibition of ovulation, the preparation may be oral, intravaginal or transdermal; progesterone-only hormonal contraception associated with inhibition of ovulation which may be oral, injectable or implantable; intrauterine device (IUD); intrauterine hormone-releasing system (IUS); bilateral tubal occlusion; vasectomised partner; sexual abstinence for 52 weeks post study treatment.

Sexual abstinence is considered to be highly effective method only if defined as refraining from heterosexual activity from the date of consent until the week 52 visit post study treatment. The reliability of this method should be evaluated in relation to the duration of the study and the preferred and usual lifestyle of the participant.
