# Supplementary material for: Intraperitoneal transplant of Hepatocytes co-Encapsulated with mesenchymal stromal cells in modified alginate microbeads for the treatment of acute Liver failure in Pediatric patients (HELP)—An open-label, single-arm Simon’s two stage phase 1 study protocol
Source: PLoS One. 2023 Jul 25;18(7):e0288185. doi: 10.1371/journal.pone.0288185 (PMC10368261; doi:10.1371/journal.pone.0288185)
Supplement: S2 File — (PDF) [file pone.0288185.s003.pdf]

Hepatocytes co-**E**ncapsulated with mesenchymal stromal cells in alginate microbeads for the treatment of acute **L**iver failure in **P**aediatric patients (HELP)

Study Acronym: HELP
Short Title: Hepatocyte Microbeads for ALF

Version 3.2 (15th Dec 2022)

Trial Identifiers:

EudraCT Number: 2019-000316-29

IRAS Number: 199623

REC Number: 22/LO/0292

ISRCTN Number: TBC

Sponsor Protocol Number: 3571

SPONSOR:

King's College Hospital NHS Foundation Trust

SPONSOR CONTACT

Rebecca Newton
King's Health Partners Clinical Trial Office, F16 Tower Wing, Guy's Hospital, Great Maze Pond, London SE19RT
T: +44 (0)20 7188 5732
F: +44 (0)20 7188 8330
E: Rebecca.newton@kcl.ac.uk

STUDY PERSONNEL

Chief Investigator:	Professor Anil Dhawan<br>Paediatric Liver Centre, King's College Hospital<br>Denmark Hill, London SE5 9PJ<br>Telephone: 0203 2994408<br>Fax: 02032994228<br>Email: anil.dhawan@kcl.ac.uk
Co-Investigator	Dr Emer Fitzpatrick<br>Paediatric Liver Centre, Institute of Liver Studies, 3rd Floor<br>Cheyne Wing, King's College London, Denmark Hill, SE5 9PJ<br>Fax: 02032994228<br>Email: emer.fitzpatrick@nhs.net
Co-Investigator	Dr Eirini Kyrana<br>Senior Lecturer Paediatric Hepatologist and Honorary consultant<br>King's College Hospital<br>Denmark Hill, London SE5 9PJ<br>Email: eirini.kyrana@nhs.net
Co-Investigator	Dr Sanjay Bansal<br>Consultant Paediatric Hepatologist<br>King's College Hospital<br>Denmark Hill, London SE5 9PJ<br>Email: Sanjay.bansal@nhs.net
Chief Scientific-Investigator	Dr Celine Filippi<br>Institute of Liver Studies, 3rd Floor Cheyne Wing, King's College Hospital, Denmark Hill, London SE5 9PJ<br>Telephone: 02032993137<br>Fax: 02032994228<br>Email: celine.filippi@kcl.ac.uk
Statisticians	Dr Abdel Douiri<br>King's College London. School of Population Health and Environmental Sciences. Addison House. Guy's Campus. London SE1 1UL<br>Telephone: 02078488224<br>Email: abdel.douiri@kcl.ac.uk

Funder	Medical Research Council

CLINICAL TRIAL PROTOCOL VERSION HISTORY

Protocol Version No	Date Issue	Details of Changes
V1.0	6 July 2020	Submitted to MHRA
V2.0	3 Sept 2020	Updated following MHRA review with addition of long term safety follow up for 10 years post IMP (including the first 52 weeks intensive follow up) and wording on tolerability and biological activity added to primary endpoints.
V3.0	19 Jan 2022	Updated with correct logo, new funder details, change in duration of contraceptive guidelines to be followed for 52 weeks post treatment, clarify control research samples and other administrative updates.
V3.1	6 May 2022	Updates made following REC initial review to clarify research samples will be identified with PIN and initials. Also, neonatal intensive added as another location for IMP infusion, correction made to clinical blood tests at follow up visits and clarified primary endpoint wording (severe).
V3.2	15 Dec 2022	Administrative correction of the definition of complete infusion on two sections, reduction in the volume of blood required for research assays, reference to HELP laboratory manual and clarification regarding prophylactic antibiotics.

Table of Content

1. Study Synopsis......7
2. Abbreviations and Glossary......10
3. Background and Rationale......12
3.1 Background......12
3.1.1 Acute liver failure in children......12
3.1.2 Hepatocyte transplantation as a treatment for ALF......12
3.1.3 Mesenchymal stromal cells enhance the function and viability of hepatocytes......13
3.1.4 Encapsulation in peptide-modified alginate......14
3.2 Rationale for intraperitoneal infusion approach......14
3.3 Summary of Hypothesis......14
4. Trial Objectives and Design......16
4.1 Trial Objectives......16
4.1.1 Primary objective......16
4.1.2 Secondary objective......16
4.2 Trial Endpoints......16
4.2.1 Primary endpoints:......16
4.2.2 Secondary endpoints:......16
4.2.3 Exploratory end points......16
4.3 Trial Design......17
4.3.1 Overview......17
4.3.2 Trial Duration and Flowchart......17
5. Trial Medication......19
5.1 HMB002 Production......19
5.2 IMP Packaging and Storage:......19
5.3 Dosing Regimen......20
5.4 Dosing Rationale......21
5.5 IMP Risks......23
5.5.1 Specific IMP risks Occurring in the first 24 hours post HMB002 infusion together with mitigating action......24
5.5.2 Occurring after first 24 hours post infusion......25
5.6 Contraindications......26
5.7 Drug Accountability and Traceability......27

5.8 Subject Compliance. 27
5.9 Concomitant Medication 27
6. Selection and Withdrawal of Subjects 27
6.1 Inclusion Criteria 27
6.2 Exclusion Criteria 28
6.3 Selection of Participants 29
6.4 Withdrawal of Participants 29
6.5 Replacement of Subjects 29
6.6 Expected Duration of Trial 29
7. Trial Procedure 30
7.1 Schedule of Assessments 30
7.2 Study Assessments by the Visit 32
7.3 Laboratory Tests 37
8. Assessment of Efficacy 37
8.1 Primary Efficacy Parameters 37
8.2 Secondary Efficacy Parameters 37
8.3 Procedures for Assessing Efficacy Parameters 38
9. Assessment of Safety 38
9.1 Specification, Timing and Recording of Safety Parameters. 38
9.2 Procedures for Recording and Reporting Adverse Events 38
9.3 Treatment Stopping Rules 41
10. Statistics 42
10.1 Sample Size 42
10.2 Analysis 42
11. Study Management 43
11.1 Trial Management Group (TMG) 43
11.2 Data and Safety Management Board (DSMB) 43
12. Ethics & Regulatory Approvals 44
12.1 Good Clinical Practice 44
12.2 Ethics Committee Approval 44
12.3 Informed Consent Procedure 44
13. Quality Assurance 45
13.1 Trial Monitoring 45

13.2 Data Handling 45
13.3 Direct Access to Source Data and Documents 46
14. Data Management 46
14.1 Data Collection 46
14.2 Source Data 47
14.3 Electronic CRF Database Access 47
14.4 Archiving 47
14.5 Publication Policy 47
15. Insurance / Indemnity 48
16. Financial Aspects 48
17. References 49
18. Appendices 50
18.1 Appendix 1 – Acute Liver Failure Classification 50

1. Study Synopsis

Title of clinical trial	Hepatocytes co-Encapsulated with mesenchymal stromal cells in alginate microbeads for the treatment of acute Liver failure in Paediatric patients(HELP)
Protocol Short Title/Acronym	Hepatocyte Microbeads for ALF / HELP
Trial Phase if not mentioned in title	I/II*
Sponsor name	King's College Hospital NHS Foundation Trust
Chief Investigator	Professor Anil Dhawan
Eudract number	2019-000316-29
REC number	22/LO/0292
Medical condition or disease under investigation	Acute liver failure in infants and children
Purpose of clinical trial	To investigate a novel treatment for acute liver failure in infants and children
Primary objective	To evaluate the safety, biological activity and tolerability of transplantation of a single dose of microbeads made from the optimum combination of peptide-alginate, mesenchymal stromal cells (MSCs) and hepatocytes in paediatric patients with acute liver failure.
Secondary objective (s)	To establish proof of concept of the transplantation of microbeads made from the optimum combination of peptide-alginate, MSCs and hepatocytes.<br><br>To inform sample size and confidence intervals to design a larger randomized clinical trial
Trial Design	Non-randomised, open-label, single-arm Simon's two stage study.
Endpoints	Primary Endpoint:<br>• Safety: Moderate to severe (including life threatening and death) adverse event occurrences due to product in 1st 52 weeks post procedure

	• Tolerability: assessed by the proportion of initiated infusions where >80% of the infusion is received by the patient.<br>• Biological activity: Patient survival with native liver at 24 weeks post treatment.<br><br>Secondary Endpoints<br>• Change in blood marker levels including haematological, biochemical and coagulation baseline to 52 weeks post treatment.<br>• Quality of life measures<br>• Patient survival with native liver at 52 weeks post treatment<br>• Patient survival with transplanted or native liver at 24- and 52-weeks post treatment<br>Exploratory Endpoints:<br>• To develop an assay 'Hepamorph' which distinguishes the recipient liver cell-derived proteins from the donor liver cell-derived proteins using a targeted mass spectrometry approach.<br>• To analyse viability and function of microbeads which are retrieved from the intraperitoneal cavity either at laparoscopy or at transplant for phenotype ex vivo.
Sample Size	Stage 1: 9 patients and Stage 2: 8 patients<br>Total 17 patients
Inclusion criteria	I. Infant or child (male or female) under the age of 16 years at recruitment.<br>II. Written informed consent obtained from a parent/legal guardian<br>III. Presence of acute liver failure (ALF) defined as a multisystemic disorder in which severe impairment of liver function with or without encephalopathy occurs in association with hepatocellular necrosis reflected as synthetic liver failure in a child with no recognised underlying chronic liver disease. Children must fit one of the ALF categories as described in Appendix 1<br>IV. Willing and able to comply with the study visit schedule
Exclusion criteria	I. Severe ascites causing high intra-abdominal pressure and / or respiratory compromise<br>II. Intra-abdominal sepsis suspected or proven

	III. Clinical condition too unstable to tolerate procedure without compromise<br>IV. Proven preexisting allergy or intolerance to alginate on medical history<br>V. Proven pre-existing allergy to gentamicin on medical history;<br>VI. Intraperitoneal or intra-abdominal malignancy<br>VII. Adhesions or fistulae to anterior abdominal wall<br>VIII. Children who weigh in excess of 33kg<br>IX. Pregnant or lactating patients<br>X. Female patients of childbearing potential who are not willing to use highly effective methods of contraception to prevent pregnancy or abstain from heterosexual activity for 52 weeks post treatment.<br>XI. Male patients who are not willing to use an effective method of contraception (condom, vasectomy, sexual abstinence) for 52 weeks post treatment, when engaging in sexual activity with a female of childbearing potential<br>XII. Participation in concurrent therapeutic trial for ALF<br>XIII. Imminent Liver transplantation expected within 12 hours of infusion<br>XIV. Total Hepatectomy<br>XV. Dependent on Extracorporeal Membrane Oxygenation (ECMO)<br>XVI. Previous liver transplant
Investigational Medicinal Product (IMP), dosage and route of administration	Total of 25 million hepatocytes per kg at a ratio of 3:1 with MSC. Infusate will consist of alginate beads (2.5 million hepatocytes per mL) plus 100% transplant medium (1:1 v/v) for suspension of the beads.<br>Single dose of IMP (HMB002) administered intraperitoneally
Active comparator product(s)	Single arm study
Maximum study duration per Subject	10 years post HMB002 infusion. This includes a 52-week intensive follow up, proceeded by a longer-term safety follow up.

* The study uses phase I/II methodology but was submitted to regulatory bodies as a phase 1 study under the MHRA definitions, as HMB002 is administered first time in humans.

2. Abbreviations and Glossary

AEs	Adverse Events
ALF	Acute Liver Failure
AR	Adverse Reaction
ASR	Annual Safety Report
ATIMP	Advanced Therapy Investigational Medicinal Product
BP	Blood Pressure
CA	Competent Authority
CI	Chief Investigator
CMV	Cytomegalovirus
Cr	Creatinine
CRA	Clinical Research Associate
CRF	Case Report Form
CTCAE	Common Terminal Criteria for Adverse Events
CTD	Common Technical Document
CTFG	Clinical Trial Facilitation Group
CTO	Clinical Trial Office
DLT	Dose Limiting Toxicity
DMC	Data Monitoring Committee
DSMB	Data and Safety Monitoring Board
DSUR	Development Safety Update Report
EBV	Epstein-Barr Virus
EC	Ethics Committee
ECM	Extracellular Matrix
ECMO	Extracorporeal Membrane Oxygenation
eCRF	Electronic Case Report Form
EDC	Electronic Data Capture
EMA	European Medicines Agency
EU-GMP	European Good Clinical Practice
EVCTM	Eudra Vigilance Clinical Trial Module
FBC	Full Blood Count
FPFV	First Patient First Visit
GCP	Good Clinical Practice
GDPR	General Data Protection Regulation
GMP	Good Medical Practice
Hb	Haemoglobin
HDU	High Dependency Unit
HE	Hepatic Encephalopathy
HMA	Head of Medicines Agencies
HSV	Herpes Simplex Virus
HTA	Human Tissue Authority
HTLV	Human T-lymphotrophic virus
IB	Investigator’s Brochure
ICF	Informed Consent Form
ICH	International Conference Harmonisation
ICU	Intensive Care Unit
IME	Important Medical Event

IMP	Investigational Medicinal Product
IMPD	Investigational Medicinal Product Do
INR	International Normalised Ratio?
IUD	Intrauterine Device
IUS	Intrauterine System
KCH	Kings College Hospital
KCL	Kings College London
KHP	Kings Health Partner
KHP-CTO	King's Health Partners Clinical Trial Office
LFTs	Liver Function Tests
MHRA	Medicine and Health Related product Authority
MSC	Mesenchymal Stroma Cells
NCI	National Cancer Institute
NHSBT	National Health Service Blood and Transplant
NIHR	National Institute for Health Research
NICU	Neonatal Intensive Care Unit
OPD	Out Patient Department
PedsQL™	Pediatric Quality of Life Inventory
PI	Principal Investigator
PICU	Paediatric Intensive Care Unit
PR	Pulse Rate
QC	Quality Control
QoL	Quality of Life
RCT	Randomised Control Trial
REC	Research Ethics Committee
RR	Respiration Rate
SAE	Serious Adverse Event
SAER	Serious Adverse Event Report
SAR	Serious Adverse Reaction
SDV	Source Data Verification
SIRS	Systemic Inflammatory Response Syndrome
SOP	Standard Operation Procedure
SUSARs	Suspected Unexpected Serious Adverse Reactions
TMF	Trial Master File
TSC	Trial Steering Committee
UAR	Unexpected Adverse Reaction
UCL	University College London
UK	United Kingdom
U&E	Urea and electrolytes
US	Ultrasound

3. Background and Rationale

3.1 Background

3.1.1 Acute liver failure in children

Acute liver failure (ALF) in children is defined as a multisystemic disorder in which severe impairment of liver function, with or without encephalopathy, occurs in association with hepatocellular necrosis reflected as synthetic liver failure in a child with no recognised underlying chronic liver disease (1).
Acute Liver Failure carries a high mortality without liver transplantation and donor organ shortage makes it difficult to provide this treatment to every liver transplant candidate in a timely fashion (2). Liver transplantation, though life-saving, also carries the risks of major surgery and complications of life-long immunosuppression. Given the tremendous regenerative potential of liver, it is possible that complete regeneration of the failing liver could be achieved post ALF. This is demonstrated in auxiliary liver transplantation, where up to 70% of recipients have shown native liver regeneration with the ability to withdraw immunosuppression leading to graft degeneration with the native liver returning to a fully functional state (3).

3.1.2 Hepatocyte transplantation as a treatment for ALF

ALF is essentially the failure of hepatocellular synthetic and detoxification function which then leads to multi-organ failure. Transplantation of hepatocytes (Liver cells rather than an organ) has been shown to improve synthetic and detoxification functions in small animal models with subsequent human application in case series of patients with ALF (4). The advantages of hepatocyte transplantation in this context are considerable. Firstly, hepatocytes are derived from organs (livers) which are otherwise unsuitable for transplantation, they can be cryopreserved and thus provide an 'off the shelf' treatment in ALF, in contrast to the wait for an appropriate organ. Secondly, the technique of hepatocyte transplantation within alginate beads which are infused into the peritoneal cavity is much less invasive than liver transplantation. Thirdly, as the alginate coating protects the cells against the body's immune system, it avoids the need for immunosuppression and associated major risks.

We have observed the safety and feasibility of intraperitoneal transplantation of hepatocytes in alginate microbeads in 8 children with ALF, with a median age of 19 days (range: 3 days to 6 years 3 months) (5). This was done not in the form of a clinical trial but on a named patient basis according to clinical need, with MHRA Specials License and institutional support. The diagnosis was Gestational Allo-immune Liver Disease (also known as Neonatal Haemochromatosis) in 4 subjects, Herpes simplex associated acute liver failure in 1 subject and indeterminate in 3 subjects. The laboratory parameters prior to treatment showed [median (range)] peak INR 4.69 (3.06 - 15), AST 215 IU/l (35-4531), ALT 49 IU/l (8-1270) and Bilirubin 144 (35-230) umol/l. All children were admitted to the intensive care unit as per standard of care. Patients received the product HMB001 which comprises primary human hepatocytes encapsulated in SLG-Alginate and suspended in transplant medium. Patients received a median of 2.4×10^7 hepatocytes / kg body weight, encapsulated in alginate microbeads and suspended in transplant medium. HMB001 was infused into the peritoneal cavity under ultrasound guidance. Two patients had a second intraperitoneal infusion (in one the initial infusion was incomplete). There were no complications associated with the infusion of beads into the peritoneal cavity. Patients were monitored in the intensive care unit until their clinical condition due to ALF was appropriate for step-down to the ward.
Four children recovered without liver transplant, and were taken off the liver transplant waiting list at a median time of 10 days (range: 7 to 31 days). All these children have now normal liver function tests.

Three underwent laparoscopic wash out of the peritoneal cavity. In two of these children there were a few strands of fibrosis within the peritoneal cavity containing beads at 3.5 months and 6 months post bead infusion respectively.
Three children were bridged to liver transplantation at day 4, day 15 and day 30 post hepatocyte transplantation. These children underwent wash-out of the peritoneal cavity at time of transplant. All three children are now well in the community. No scarring or other complications were observed at the time of liver transplant operation.
One child, despite stabilisation of his liver tests had further multi-organ clinical deterioration and care was withdrawn due to underlying diagnosis of Trisomy 21 and severe cardiac failure owing to a congenital cardiac abnormality.
Laboratory analysis of the alginate beads, retrieved from the peritoneal cavity showed some viable hepatocytes with preserved synthetic and detoxification function at a maximum interval of 6 months and 9 days.
Thus, the safety of the procedure has been established by this named-patient intervention under a MHRA Specials licence with no adverse events attributed to the intraperitoneal infusion of hepatocyte microbeads. These children all remain under surveillance. The finding of strands of fibrosis at wash out in two patients after 3 and 6 months has prompted us to undertake laparoscopy prior to discharge of the patient from hospital and usually within 1 month of the microbead infusion.
Children were followed up for 7 years. Though expected survival without transplantation is 10% - 20% in this group, 50% (4 children) survived with their native liver and 3 (37.5%) were successfully bridged to transplant. This is particularly important for infants and small children in whom the wait for an appropriately sized organ, most often from a size matched donor may be extremely prolonged.
However, there are some limitations of the technique. This is predominantly the relatively restricted availability of good quality hepatocytes and the ability of the cells to survive and function well in the intraperitoneal cavity for a number of weeks.

3.1.3 Mesenchymal stromal cells enhance the function and viability of hepatocytes

Our previous work and that of others has demonstrated that co-culture of hepatocytes with mesenchymal stromal cells (MSC) dramatically improves their survival and function in vitro, although the mechanism of this effect is yet to be fully described. MSC are known to improve tissue repair, through localised immune-suppressive effects and the release of soluble trophic factors. These properties make them excellent candidates for improving the survival of transplanted cells (6), as shown in hepatocyte cultures, animal models of liver disease and pilot clinical studies of ALF. Thus, MSC effects on both the injured native liver and on co-encapsulated hepatocytes could prove to be an attractive novel therapy. Soluble factors such as growth factors, cytokines, extra-cellular matrix glycoproteins and other small molecules produced by MSC possibly mediate these effects (7). This anti-apoptotic, pro-regenerative effect of MSC has also been seen in the setting of myocardial infarction (8) and stroke (9). In previous work we have demonstrated that the human MSC derived from adipose tissue and umbilical cord improve hepatocyte-specific functions of co-cultured hepatocytes. In particular, albumin secretion by hepatocytes cultured at a 3:1 ratio with MSC was 10-fold higher than that by hepatocytes in monoculture by day 15. This effect was still seen at day 25. This improvement was seen best in direct co-culture but is also seen in indirect co-culture, where cells were separated with a trans-well insert, indicating the possible contribution of trophic factors secreted by the mesenchymal stromal cells. We have also found that total cell death (and specifically hepatocyte apoptosis) was decreased in both direct and indirect co-cultures. There was also a decrease in apoptosis in hepatocytes cultured in MSCs conditioned medium after 7 days (6). This effect was more pronounced in conditioned medium from co-culture versus MSCs monoculture suggesting that MSCs need to be activated by hepatocytes to produce an optimal effect.

3.1.4 Encapsulation in peptide-modified alginate

We have found that the interaction of hepatocytes and MSC in the standard GMP SLG-alginate preparation is not as effective in improving hepatocyte function as when the cells are in direct contact. We hypothesise that this is due to poor anchoring of cells to the surrounding extracellular matrix and that use of modified ultrapure alginate preparations with different anchorage peptides will surmount this obstacle. The main hypothesis of the proposed study is that co-encapsulation of hepatocytes with MSC, in alginate microbeads which allow the appropriate cell anchorage, will substantially improve the survival and function of hepatocytes and be a feasible and effective form of cellular therapy for acute liver failure.

3.2 Rationale for intraperitoneal infusion approach

Intraperitoneal infusions are a very common practice and dialysis for renal failure is an example with long term safety record. Peritoneal puncture is a very common procedure that is carried out in the liver wards for ascitic tap. The procedure is carried out with local infiltration of the skin with local anaesthetic. No adverse events like bleeding, intestinal perforation or infection were observed in any of the patients treated to date. The animal data and the data from our longest follow up over 3 years have not shown any untoward events. Laparoscopic examination in the children who have survived with native liver has shown no significant scarring in the peritoneal cavity. Subsequent liver transplantation in 3 children did not pose any difficulties at the time of surgery or any complications post liver transplantation that could be attributed to alginate bead transplantation.

Our current alginate encapsulation technique uses PRONOVA™ UP, an ultrapure GMP-produced alginate that satisfies the highest ISO standards. We have optimised the alginate percentage, cell density and bead size allowing the best cell survival, and recently demonstrated that this alginate is not immunogenic in our in vitro tests (10). However, this alginate appears to be less effective in providing anchorage, leading to lower cell survival and cell division capacity. In the case of primary hepatocytes, when isolated from their niche, i.e. from their various stromal cells and extracellular matrix (ECM), they de-differentiate, losing 50% of albumin synthesis capacity within 1-2 days. Culture of cells on a mix of alginate and natural ECM components improves cell viability and function, whilst natural or synthetic matrices bound to specific anchoring motifs dramatically improves cell attachment, preventing cell death and loss of function.

3.3 Summary of Hypothesis

The use of co-encapsulated hepatocytes and mesenchymal stromal cells in the optimal formulation of alginate is a safe and effective form of liver support in paediatric acute liver failure. Though the safety and possibly short-term efficacy of encapsulated hepatocytes alone has been observed in a named patient use by us to date, the medium term (up to 24 weeks while awaiting liver regeneration) success of the cell therapy has not been achieved. Thus, our goal is optimisation of hepatocyte function and viability using modifications of the alginate and MSC co-encapsulation. This study will involve intra-peritoneal infusion of HMB002 which are co-encapsulated hepatocytes and mesenchymal stromal cells at a ratio of 3:1 at a dose of 25 million hepatocytes per kg. Infusate will consist of alginate beads (2.5million cells per mL) plus 100% transplant medium (1:1 v/v) for suspension of the beads. This is given as a once off infusion under ultrasound guidance in addition to standard of care. The beads will be removed using laparoscopy prior to discharge of the patient from hospital or at time of transplantation to minimise or eliminate risk of adhesions within the peritoneal cavity. The other potential adverse events are an increase in intra-abdominal pressure due to the volume of beads and medium. This will be closely monitored at time of infusion and in cases of significant ascites, infusion

may not be possible. The expected outcome using this product is that the infant or child may be bridged to either recovery of the native liver or to the time when a suitable organ becomes available for liver transplantation.

4. Trial Objectives and Design

4.1 Trial Objectives

4.1.1 Primary objective

To evaluate the safety, biological activity and tolerability of transplantation of a single dose of microbeads made from optimum combination of peptide-alginate, mesenchymal stromal cells (MSC) and hepatocytes to paediatric patients with acute liver failure.

4.1.2 Secondary objective

To establish proof of concept of the transplantation of microbeads made from the optimum combination of peptide-alginate, MSC and hepatocytes.

To inform sample size and confidence intervals to design a larger randomized clinical trial (RCT).

4.2 Trial Endpoints

4.2.1 Primary endpoints:

- Safety: Moderate to severe (including life threatening and death) adverse event occurrences due to product in 1st 52 weeks post procedure
- Tolerability: assessed by the proportion of initiated infusions where >80% of the infusion is received by the patient.
- Biological activity: Survival with native liver at 24 weeks post treatment.

4.2.2 Secondary endpoints:

- Change in blood marker levels including haematological, biochemical and coagulation baseline to 52 weeks post treatment.
- Quality of life measures
- Patient survival with native liver at 52 weeks post treatment
- Patient survival with transplanted or native liver at 24- and 52-weeks post treatment.

4.2.3 Exploratory end points

- To assess the assay 'Hepamorph' which distinguishes the recipient liver cell derived proteins from the donor liver cell derived proteins using a targeted mass spectrometry approach.
- To analyse viability and function of microbeads which are retrieved from the intraperitoneal cavity either at laparoscopy or transplant for phenotype ex vivo.

4.3 Trial Design

4.3.1 Overview

This is an, open-label, single-arm, single centre study. A Simon's two-stage design for a one-sample exact test will be used. We assume a one-year survival rate of 0.20 or less under the null hypothesis, and 0.5 or more under the alternative, with 80% power and 5% type I error rate.

The study will be conducted in two stages. Nine patients will be recruited during stage 1 of the study. Once 9 patients have completed their 24 weeks visit, the study will stop for futility if only 2 or fewer patients have survived with the native liver. Otherwise, DSMB approval is required for progression to Stage 2, where the trial will continue to recruit a further 8 patients. A total of 17 patients will be recruited into the study (at the end of stages 1 and 2). At the end of the second stage, if 7 or more patients out of the 17 enrolled have survived with native liver at 24 weeks post HMB002 treatment, this will demonstrate proof of concept and would support the design of a larger RCT.

The trial will also report 52-week survival outcome, this is not anticipated to change from 24-week outcome as this trial is to attempt to rescue acute liver failure. If the 52-week outcome differs from the 24-week outcome this observation will be an important consideration in the design of any future trial. A long term follow-up period will be used to monitor safety over a 10 year period post IMP infusion.

The primary outcome survival measure is set at 24-weeks. Although response to treatment is anticipated to be within the first 4 – 6 weeks, short to medium term morbidity in children who have been critically unwell in the Paediatric Intensive Care Unit (PICU), Neonatal Intensive Care Unit (NICU) or High Dependency Unit (HDU), is best determined at a longer time point such as 24 weeks. This way almost all possible later deaths due to the acute liver failure (or early post-transplant deaths) will be effectively captured.

4.3.2 Trial Duration and Flowchart

Total Estimated Duration from FPFV:
We will aim to recruit the 17 patients (stage 1 and 2) in approximately 36 months. Each individual patient will be followed up in the trial for 10 years after receiving HMB002 infusion. Patients will be intensively followed up for 52 weeks and then for long term safety monitoring for a further 9 years.

The end of the trial will be defined as the last patient 10 year annual follow up post IMP infusion. The duration of the study will vary dependent on whether the study progresses to stage 2 or is stopped for futility following stage 1 Go/No Go DSMB review.

Trial Flowchart for Screening and Recruitment

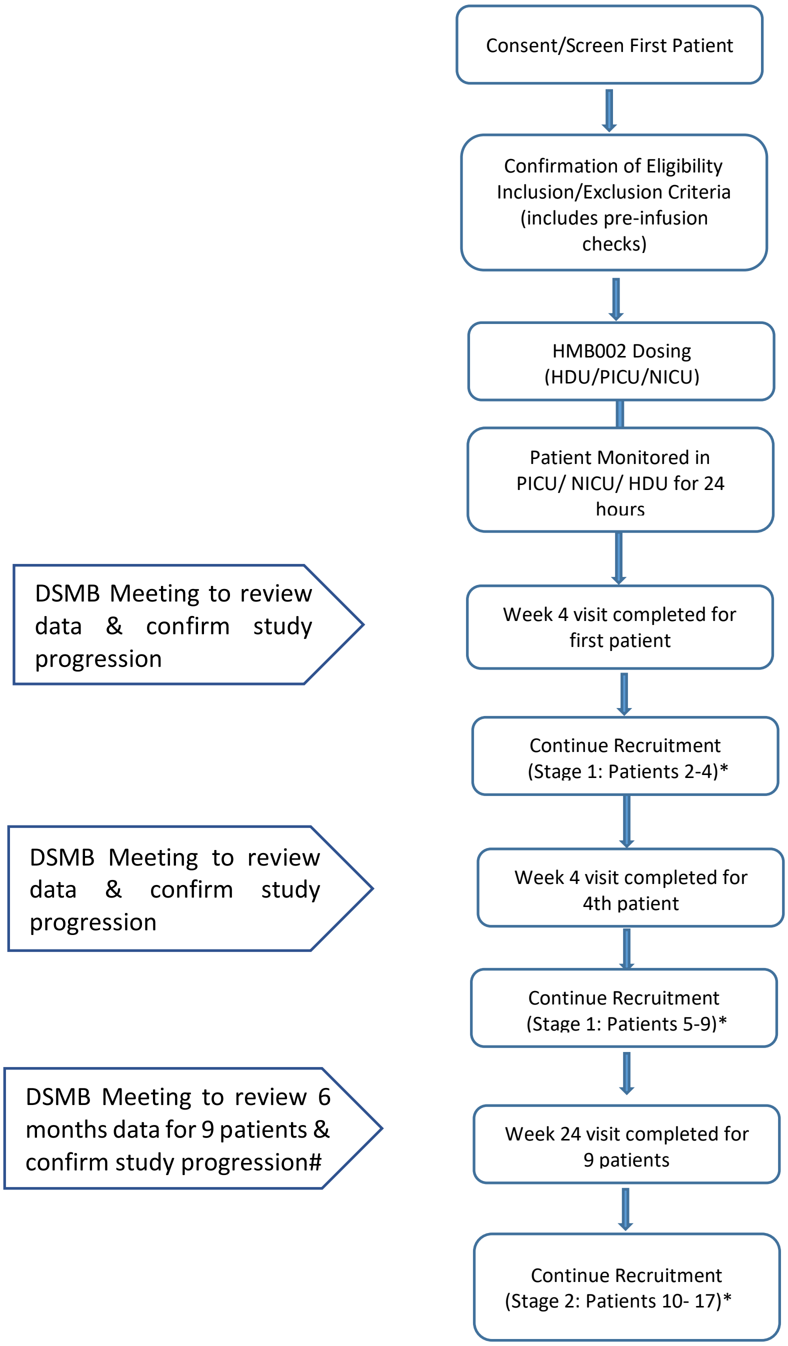

*Interim DSMB meetings as advised by DSMB Chair for the duration of the study.
Study will be terminated if 2 or fewer patients survive with native liver at 24 weeks post HMB002 infusion, after stage 1 of the trial.

A total of 17 patients will be recruited into the study (stage 1= 9 patients and stage 2 = 8 patients).

5. Trial Medication

5.1 HMB002 Production

The final investigational product will be made of:

(i) primary human hepatocytes isolated from donor organs, procured through NHSBT after specific consent for liver cell isolation, in the NIHR/Welcome Trust Cell Therapy Unit at King's College Hospital (HTA Licence Number 11062)

(ii) human umbilical cord mesenchymal stromal cells (MSC) procured by the Anthony Nolan Trust with consent and isolated in King's College Hospital GMP Cell Therapy Unit above (under specials licence MS14523).

(iii) a peptide alginate MVG GRGDSP in which the cells are encapsulated (Dupont)

(iv) transplant medium (CMRL 1066 with Human Albumin Solution).

The final product is named as HMB002. The alginate gel microbeads obtained from the encapsulation protect the cells from the recipient immune system whilst allowing oxygen and circulating toxins to reach the cells, and products of cell function—e.g. proteins, blood detoxification products—to be secreted and reach the blood stream when implanted into the peritoneal cavity.

The alginate microbeads will be manufactured in a GMP unit, from cells isolated/prepared and cryopreserved following EU-GMP regulation. For full details, see the Investigational Medicinal Product Dossier (IMPD) for the study. Briefly, the alginate is reconstituted from lyophilised powder, before being mixed with the cells: $2.5x10^6$ hepatocytes/mL and 0.83 $x10^6$ MSCs/mL of alginate, after QC check of each respective cell suspension. The cells are then encapsulated using an encapsulator (e.g. Buchi B395 Pro), producing a jet of micro-droplets then crosslinking/jellifying into a bath of 0.1M calcium chloride. After crosslinking, the beads are rinsed in saline twice, before being resuspended in transplant medium. The final product ($25x10^6$ hepatocytes/kg and $8.3x10^6$ MSC/kg in 10mL/kg alginate and 10mL/kg transplant medium) is packaged as 20mL/kg final volume in sterile syringes for infusion, once QC checks are satisfactory. Children who weigh in excess of 33kg cannot be treated within the study due to current capacity for HMB002 manufacture. Children with a known allergy to gentamicin will be also be excluded from the trial as MSCs are manufactured using gentamicin.

The donation, procurement and testing of the human tissues and cells are in conformity with the relevant Regulations, as referred to in Article 3 of the Regulations (EC) 1394/2007. The donation of the human cells used to manufacture the IMP is not considered a part of this trial.

5.2 IMP Packaging and Storage:

The final product (HMB002) is packed in sterile syringes and kept at 2 to 8°C with an icepack from time of manufacture to administration. The IMP will be administered shortly after production and always within 8 hours from time of manufacture. Release criteria for the final product are detailed in the IMPD, and in the table below. The release will be split in two phases, an interim release and a final release depending on the time necessary to get each of the test results. Some tests can be obtained prior or shortly after the microbead production (all data on cryopreserved cells, microbead size, endotoxin concentration and gram stain) whilst others require days to weeks to be processed (mycoplasma, sterility). The treating physician will be informed if the product fails any of the final release tests.

Table 1: QP release specifications

Parameter	Interim / final release
Cryopreserved Hepatocyte QC data	Interim
Cryopreserved MSC QC data	Interim
Bead size data	Interim
Endotoxin	Interim
Gram stain	Interim
In process control – cell sterility using BacTAlert system	Final (for information)
Mycoplasma	Final
Final product sterility testing	Final

Each syringe will be labelled following EU-GMP Annex 13. The product package will also be labelled and will be placed on cool-packs inside a cool box and must be accompanied by the completed release form.

5.3 Dosing Regimen

The route of administration of the product is intra-peritoneal. A single dose (weight-dependent) will be used in all patients. As this is a single infusion there are no increments of dosage. The dose will be administered as soon as possible following parental/legal guardian consent and screening of the patient, as these patients are critically ill as per inclusion criteria. The infusion will be administered at a rate of approximately 150-200ml per hour and close monitoring will be undertaken. This dose has been determined using in vitro, in vivo and previous clinical pilot experience, as detailed in the IMPD and Investigator Brochure (IB). The target population is too small and too unwell to do any significant dose escalation study. Any further dose discovery studies in healthy volunteers could not be justified. There are no special dietary or other requirements from the participants. They will have standard of care treatment and monitoring involving adherence to standard treatment regime and receive immunosuppressive treatment in the case of liver transplantation.

As this is a first-in-human study, the first patient will undergo HMB002 infusion followed by a 4-week period of monitoring prior to the recruitment of a second patient. The study will be overseen by a data and safety monitoring board (DSMB). The DSMB will be convened to review safety data from the first patient prior to recruiting the second patient. No patient will be treated within 48 hours of another patient and recruitment will continue as and when suitable patients present with acute liver failure. DSMB will reconvene again to review safety data following a 4-week period of monitoring after the fourth patient.

Dosing will continue until end of stage 1 (9th patient receives IMP infusion). DSMB will review 24-week safety data at the end of stage 1. Following a GO/NO GO decision from the DSMB, recruitment and dosing will continue to stage 2 of the study, where a further 8 patients will be enrolled into the trial (as outlined in Figure 1). DSMB will convene to review safety data and approve progression of the study, at intervals detailed in the DSMB charter. DSMB may also decide on next point of their review as required.

5.4 Dosing Rationale

The dosing rationale comes from original preclinical studies performed for the use of HMB001 microbeads (made of alginate and hepatocytes, without any MSC) both *in vitro* and *in vivo,* to investigate the optimal hepatocyte density. To this aim, alginate microbeads containing an increasing number of hepatocytes from $2.0x10^6$ to $3.5x10^6$ cells/mL were produced. Urea and albumin production, two common markers of hepatocyte functions, did not show any significant difference between the groups analysed on either day 1 or day 3. However, cytochrome P450 1A1/1A2 activity on day 3 was significantly higher in the microbeads containing 2.0 and 2.5×10^6 cells/mL than in the other conditions. Therefore, microbeads containing $2.5x10^6$ hepatocytes/mL were used for preclinical *in vivo* studies performed in an animal model of acute liver failure (Sprague Dawley rats treated with D-galactosamine). Each animal received an intraperitoneal injection of microbead suspension at a dose of 10 mL/kg, in line with the guidelines for acceptable injection volumes in rodents. This dose was proven effective to treat acute liver failure, with a 72-hour survival rate of 100% in the group treated with hepatocytes microbeads (10).
The same dose was proven safe when the same microbeads were used in paediatric patients with acute liver failure, treated on a named-patient basis (5).

Additional preclinical *in vitro* and *in vivo* studies showed that the co-encapsulation of human hepatocytes and mesenchymal stromal cells (MSC) in alginate microbeads significantly improved hepatocyte functions when a ratio of 3:1 (hepatocyte : MSC) was used. Therefore, a dose of $25x10^6$ hepatocytes and $8.3x10^6$ MSC per kg of body weight will be used in the proposed clinical trial. Infusate will consist of alginate microbeads (2.5 million hepatocytes and 0.83 million MSC per mL of alginate; 10mL/kg) plus 1:1 transplant medium to resuspend the microbeads, as optimised in the preclinical work.

An infusion of more than 80% of the final product for the patient will be considered as a completed infusion.

Table 2: List of Studies Investigating the Pharmacology of Alginate encapsulated cells.

Study Number	In vitro/In vivo/ Cells/Species/Strain	Dose/Treatment/Route/Duration	Endpoint Measurement	Noteworthy Findings	GLP
08/H0808/41_ VI39	In vitro<br>Cells: Human primary hepatocytes (HC186).	2.5x10^6/ml HC in SLG_{20} vs MVG GRGDSP alginate microbeads.	• Cell viability, i.e. FDA/PI staining (day 9).	HC encapsulated in MVG GRGDSP alginate microbeads showed overall higher viability compared to SLG_{20} 9 days after encapsulation.	No
08/H0808/41_ VI53/VI57/VI59	In vitro.<br>Cells: Human primary hepatocytes (HC343, HC349, HC353, HC364); umbilical cord derived MSC (SW0G, VI2, SW4).	2.5x10^6/ml HC ± 0.83x10^6/ml MSC encapsulated in SLG_{20} vs MVG GRGDSP alginate microbeads. Microbeads cultured up to 14 days.	• Cell viability, i.e. Calcein-AM metabolism (day 1, 3, 7, 14);<br>• Albumin production (day 1, 3, 7, 14);<br>• α1-antitrypsin production (day 1, 3, 7, 14);<br>• Urea production (day 1, 3, 7, 14)<br>• Phase 1 activity: Cyp1A1/1A2 activity (day 4, after induction with 50µM omeprazole for 72 hours); Cyp3A4 activity (day 4, after induction with 25µM rifampicin for 72 hours);<br>• Phase 2 activity, i.e. resorufin conjugation (day 4).	Cell viability was similar when HC were encapsulated in SLG_{20} or MVG GRGDSP alginate microbeads, however there was a tendency towards higher viability when HC were co-encapsulated with MSC in MVG GRGDSP alginate microbeads (i.e. HMB002).<br>The release of human albumin and α1-antitrypsin in cell culture medium at day 7 and 14 was significantly higher when HC were co-encapsulated with MSC in MVG GRGDSP alginate (i.e. HMB002) compared to HC in SLG_{20} (i.e. HMB001).<br>The activity of Cyp1A1/1A2 was significantly induced only in HMB002.<br>No differences were noticed in terms of urea production, Cyp3A4 induction and phase 2 activity in the groups analysed.	No
08/H0808/41_ VI39	In vivo<br>Cells: human primary hepatocytes (HC186); umbilical cord derived MSC (VI2).<br>Animals: Sprague Dawley rats	Alginate microbeads: 2.5x10^6/ml HC ± 0.83x10^6/ml MSC encapsulated in SLG_{20} vs MVG GRGDSP alginate microbeads. SLG_{20} empty microbeads as a control.<br>In vivo transplantation: cell alginate microbeads were resuspended in transplant medium (2:1 v/v) and intraperitoneally transplanted in rats as a single dose (10ml microbeads/kg). Negative controls: transplant medium (sham) and SLG_{20} empty microbeads.	• Human albumin released in bloodstream (day 1, 3, 7, 14, 28);<br>• Human α1-antitrypsin released in bloodstream (day 1, 3, 7, 14, 28).	Human albumin and α1-antitrypsin levels detected at day 1, 3 and 7 in the plasma of rats transplanted with HMB002 were significantly higher than the levels measured in the animals transplanted with HMB001. No human proteins were detected afterwards.<br>At day 7 after transplantation, the level of human albumin detected seems to be increasing compared to earlier time points, though not reaching any statistical significance (99±25 vs 68±6 ng/ml at day 7 and 1, respectively).	No

5.5 IMP Risks

Risk Assessment

Of note the patients in this study will all be suffering from acute liver failure and so deterioration and death are expected within 48 – 72 hours without any treatment, regardless of HMB002 infusion. The risk-benefit profile of this clinical trial essentially balances with the clinical benefits of preventing death due to acute liver failure while bridging the child to organ availability for transplant or to spontaneous recovery of the native liver. The children enrolled will all meet listing criteria for liver transplant in terms of severity of disease but in some cases liver transplant may be contraindicated i.e. they may have multi-systemic disease which would not be cured by transplantation. These children will otherwise die in 90% of cases.

As HMB002 has never been tested in humans before, there is currently no available list of expected medical events / reactions. Hence any serious events that are deemed related to HMB002 (serious adverse reactions or SARs) will be considered suspected unexpected serious adverse reactions (SUSARs). Procedures for monitoring, recording and reporting of adverse events are described in the trial protocol (section 9). The following measures will be taken to ensure that the maximum safety for subjects participating in the trial is assured:

- Continuous monitoring in Paediatric Intensive care unit or high dependency unit (HDU) prior to the infusion and for at least 24 hours post infusion.
- Standard care includes use of prophylactic antibiotics and antacids.
- Cardiovascular and respiratory stability confirmed prior to infusion (this may involve ventilation and inotropic support).
- Correction of coagulopathy and ensuring adequately stable haemoglobin and electrolytes prior to infusion.
- Regular and thorough patient monitoring following infusion (vital signs, clinical examination, blood tests) as both an inpatient and later as an outpatient
- Ensuring that HMB002 are washed out prior to discharge from hospital following recovery with either native liver or post liver transplant.

A detailed list of all possible IMP risks as part of the trial is provided below.
AEs will be graded according to Common Terminology Criteria for Adverse Events (CTCAE) criteria.

Expected complications of acute liver failure include coagulopathy, hypoglycaemia, sepsis, encephalopathy, renal dysfunction and failure, circulatory failure, raised intracranial pressure and a systemic inflammatory reaction syndrome (SIRS) which occurs with massive cytokine release or from other factors from the damaged liver.

PICU/NICU support includes ventilatory support, ionotropic support, renal replacement therapy, intracranial pressure monitoring and treatment with position, hypercarbia, hypertonic saline and mannitol. Occasionally hepatectomy may be required while an organ is awaited. Extracorporeal membranous oxygenation (ECMO) has also been used at our institution for children with multi-organ failure as a consequence of fulminant liver failure. We anticipate that children who have undergone total hepatectomy or are on ECMO will be too unstable to tolerate the proposed intervention and thus will be excluded from the trial.

5.5.1 *Specific IMP risks Occurring in the first 24 hours post HMB002 infusion together with mitigating action*

Bleeding from puncture site or intraperitoneal bleeding

This may occur in a coagulopathic patient though the bleeding risk in patients with acute liver failure is considerably lower than their clotting parameters would infer. Children may need blood products prior to the procedure in order to correct their INR to <2 (though this may not be achievable), platelet infusion should be given if platelet count is <50,000 per µL and fibrinogen should be >1.0 g/L. Haemoglobin should be >7 g/dL prior to the procedure.
The infusion will be undertaken under ultrasound guidance and an additional ultrasound will be undertaken if there is any suspicion of intraperitoneal bleeding. As no major vessels should be punctured, it is expected that any such bleeding should be controlled using correction of coagulopathy. Both interventional radiology and surgical expertise are available in house to manage bleeding should it persist.

Rise in intra-abdominal pressure and cardiovascular compromise

If HMB002 is infused in such quantities in the presence of ascites, there is a risk that intra-abdominal pressure may rise and compromise respiratory function. It may be possible to monitor intra-abdominal pressure will be monitored pre and post HMB002 infusion, using an indwelling urinary catheter. This may however not be possible in very young patients or if the risks outweigh the benefits. Close attention will be made of the intraabdominal pressure change and any compromise in respiratory status. The measurement in kPa is only one element of assessment of intra-abdominal pressure. Other features include oliguria and severe respiratory compromise, both due to pressure effects. The infusion will be terminated in the situation that this were to occur.

Inflammatory / immunological reactions

Though alginate is bioinert and has not shown any deleterious effect in animal models, it is not possible to rule out unintended immediate inflammatory / immunological reactions to HMB002 such as cytokine release or local inflammatory response causing fluid accumulation and adhesions in the long term. The child will be monitored continuously in PICU/NICU/HDU for at least 24 hours post infusion. Steroids may be required for reversal of any immunological reactions.

Type 1 hypersensitivity reactions.

Antigenic challenge can drive IgE production leading to activation and degranulation of mast cells and basophils and the release of vasoactive, spasmogenic mediators and proinflammatory cytokines. Clinical manifestation ranges from the mild urticarial to severe anaphylactic shock. The possibility of hypersensitivity reactions against the alginate beads or HMB002 or excipients cannot be discounted entirely but the fact that alginate is a bioinert material means that the risk is less likely. Patients will be admitted to PICU/ NICU/HDU for the infusion and closely monitored for signs of reactions. Patients will be under continuous monitoring in PICU/NICU/ HDU for 24 hours at least post infusion, thus such reactions would be immediately recognised and treated as appropriate with antihistamines, steroids, and intramuscular adrenaline as required.

Nonspecific adverse immunological reactions

Release of cytokines upon infusion of the product can cause febrile reactions. This is less likely with intraperitoneal infusion but cannot be excluded. Mild febrile responses do not constitute

a major clinical concern. As above, children will be monitored continuously and will be inpatients for more than a week post infusion in all cases. They will be treated with prophylactic antibiotics in any case (as per King's College Hospital paediatric acute liver failure microbiology guidelines).

Infection

Local infection

Though the procedure will be undertaken with sterile precautions, there is a possibility of local site infection and cellulitis which will be anticipated and treated early with antibiotics.

Intraperitoneal infection

Though the procedure will be undertaken using sterile precautions there is a possibility of intraperitoneal infection being introduced during infusion. Patients will be treated with prophylactic antibiotics as standard of care. In the case that infected ascitic fluid or an infected intra-abdominal collection is suspected, a diagnostic aspiration will be performed by radiology to guide treatment and appropriate antibiotics will be used for treatment.

Bacterial infectious risk for product mitigation

Gram stain is used as a point of care test for the product, but full sterility testing of the final product will only be available 2 weeks after IMP infusion. Though all procedures involving the final product will be conducted in an aseptic manner, prophylactic antibiotics will be given to the patient for 48 hours in any case, as per King's College Hospital paediatric acute liver failure microbiology guidelines.

Other infection from product

Donors from whom cells are isolated are routinely tested for viral infection prior to use of the cells. Donor cells positive for Hepatitis B, Hepatitis C, HIV and HTLV are not used in the manufacture. EBV, CMV and toxoplasmosis are also tested in the donor as per NHSBT guidelines. Cells from CMV and EBV positive donors may be used as per standard transplant practice. The prevalence of EBV and CMV positivity (IgG) in the donor population is high. Immune suppression is not used with the infusion of HMB002 so the risk of significant infection from either EBV or CMV is low. Cells from toxoplasmosis positive patients will not be used.

5.5.2 Occurring after first 24 hours post infusion

Adhesions

Long term (>6 months) intraperitoneal exposure to HMB001 has shown that adhesions may develop in the intra-abdominal cavity if not removed (5). Potentially this could give rise to bowel obstruction and other complications depending on the site of the adhesion. This side effect is likely mediated by break down of beads and release of relatively immunogenic hepatocytes over time. In order to counteract the risk of adhesions, all children will have the microbeads washed out laparoscopically or removed at time of transplant, prior to discharge from hospital. The timing of this is not possible to predict as it will depend on the time taken for the patient to recover to normal or near normal native liver function, or the time it takes to find a suitable organ for transplantation if the native liver does not recover. Usually this will be within 4 weeks of the procedure but in any case, microbeads should be retrieved within 24 weeks of HMB002 infusion. Adhesions are usually managed conservatively however surgical intervention is sometimes necessary.

Recurrence of acute liver failure.
Children rarely have recurrence following presentation with acute liver failure however this may occur in certain circumstances (recurrent acute liver failure syndromes). There is a small possibility that if a child with recurrent acute liver failure is treated with HMB002 and recovers, that the acute liver failure may occur at a later time point and after wash out of beads. In this case, children will be treated for ALF as standard of care.

Complications relating to laparoscopic wash out
Anaesthetic complications
Complications include; standard respiratory, circulatory complications relating to anaesthetic agents and intubation. PICU is fully equipped and adequately staffed to manage all relevant emergencies.

Bleeding from laparoscopic port site
In order to prevent this, children who have abnormal clotting parameters will be corrected as standard though we do not anticipate significant abnormalities at this point post recovery (beads are washed out at time of full recovery). Rarely, laparoscopy needs to be converted to laparotomy to control bleeding.

Local or intraperitoneal infection
Laparoscopy is a sterile procedure and operative infection is rare. Antibiotic prophylaxis will be given peri-operatively as per Trust guidelines as this is a group vulnerable to infection.

Laparoscopic injury to organs during wash out.
Laparoscopy will be conducted by an experienced surgeon in laparoscopic techniques. Wash out of the peritoneal cavity is a relatively minor procedure but all precautions are taken as standard to prevent secondary complications. Rarely laparoscopy needs to be converted to laparotomy to control injury.

5.6 Contraindications

HMB002 safety information does not currently exist but the following are considered contraindications for this therapy:

- Severe ascites causing high intra-abdominal pressure
- Intra-abdominal sepsis suspected or proven
- Clinical condition too unstable to tolerate procedure without compromise
- Proven allergy or intolerance to alginate on medical history
- Proven pre-existing allergy to gentamicin on medical history (as MSC are manufactured using gentamicin)
- Intraperitoneal or intra-abdominal malignancy
- Adhesions or fistulae to anterior abdominal wall
- Pregnancy
- Participation in concurrent therapeutic trial for ALF
- Imminent liver transplantation expected (within 12 hours of infusion)
- Total hepatectomy
- Patient dependent on extracorporeal membranous oxygenation

5.7 Drug Accountability and Traceability

Full accountability will be maintained. The IMP order form will serve as the prescription for each patient. Each IMP dose will have a “batch manufacturing record” and a “release certificate”, which will serve as the IMP accountability for the purposes of the trial. Once used, vials of IMP will be immediately destroyed and will not be returned to the production facility. If the product is unused or in part unused it will be returned to the Cell Therapy Unit for record keeping, and will be appropriately disposed of according to the relevant standard operating procedure (SOP), once authorised by the CI.

5.8 Subject Compliance.

Not applicable for IMP as this will be administered by the study team. Administration of IMP, study visits and blood tests will be recorded on the electronic case report form (eCRF).

5.9 Concomitant Medication

All children will be treated as per standard protocol for acute liver failure. This may vary according to the actual or suspected aetiology of the condition but will usually include:

- Neuroprotection with ventilation, positioning, sedation.
- Cardiovascular support using inotropes,
- Haemofiltration for hyperammonaemia, oliguria and / or renal dysfunction
- Antibiotics and antifungals
- Other medications: N-acetyl cysteine, ranitidine / proton pump inhibitor or similar, intravenous vitamin K.
- Blood products: red cells, platelets, fresh frozen plasma, cryoprecipitate

Data will be collected relating to concomitant medications at every study visit (outlined in the schedule of assessments section 7.0). A complete listing of all concomitant medication received during the treatment phase must be recorded in the relevant eCRF.

6. Selection and Withdrawal of Subjects

6.1 Inclusion Criteria

I. Infant or child (male or female) under the age of 16 years at recruitment.
II. Written informed consent obtained from a parent / legal guardian;
III. Presence of ALF defined as a multisystemic disorder in which severe impairment of liver function with or without encephalopathy[a] occurs in association with hepatocellular necrosis reflected as synthetic liver failure in a child with no recognised underlying chronic liver disease. Children must fit one of the ALF categories as described in Appendix 1[b];
IV. Willing and able to comply with the study visit schedule.

Foot note:
[a] Diagnosis of encephalopathy may not be possible in infants and small children
Other parameters become more relevant

[b] Children who meet inclusion criteria as above but would otherwise not be suitable for liver transplant because of progressive neurological disease for example, will not be excluded from the trial unless they also have exclusions as detailed in criteria for the trial.

6.2 Exclusion Criteria

I. Severe ascites causing high intra-abdominal pressure and / or respiratory compromise;
II. Intra-abdominal sepsis suspected or proven;
III. Clinical condition too unstable to tolerate procedure without compromise;
IV. Proven pre-existing allergy or intolerance to alginate on medical history;
V. Proven pre-existing allergy to gentamicin on medical history;
VI. Intraperitoneal or intra-abdominal malignancy;
VII. Adhesions or fistulae to anterior abdominal wall;
VIII. Children who weigh in excess of 33kg
IX. Pregnant or lactating patients (positive pregnancy test for females of child bearing potential at screening).
X. Female patients of childbearing potential who are not willing to use highly effective methods of contraception to prevent pregnancy or abstain from heterosexual activity for 52 weeks post treatment.
*Females of child bearing potential are females who have experienced menarche and are not surgically sterilised (e.g. by tubal occlusion, hysterectomy, bilateral salpingectomy) or post-menopausal (defined as at least 1 year since last regular menstrual period).
** Highly effective methods of birth control are those with a failure rate of < 1% per year when employed consistently and correctly.
Highly effective methods of contraception as per HMA / CTFG working group are combined (oestrogen and progestogen containing) hormonal contraception associated with inhibition of ovulation, the preparation may be oral, intravaginal or transdermal; progesterone-only hormonal contraception associated with inhibition of ovulation which may be oral, injectable or implantable; intrauterine device (IUD); intrauterine hormone-releasing system (IUS); bilateral tubal occlusion; vasectomised partner; sexual abstinence for 52 weeks post study treatment;
*** Sexual abstinence is considered to be highly effective method only if defined as refraining from heterosexual activity from the date of consent until the week 52 visit post study treatment. The reliability of this method should be evaluated in relation to the duration of the study and the preferred and usual lifestyle of the participant.
XI. Male patients who are not willing to use an effective method of contraception (condom, vasectomy, sexual abstinence) for 52 weeks post study treatment, when engaging in sexual activity with a female of childbearing potential;
XII. Participation in concurrent therapeutic trial for ALF;
XIII. Imminent liver transplantation expected within 12 hours of infusion;
XIV. Total hepatectomy;
XV. Dependent on Extracorporeal Membrane Oxygenation (ECMO);
XVI. Previous liver transplant

6.3 Selection of Participants

Patients will be recruited from the Paediatric Liver Centre King's College Hospital, one of 3 centres in the UK to which all children with acute liver failure will be referred. All study visits up to 52 weeks will be conducted at King's College Hospital (KCH). Travel costs will be covered for both patient and legal guardian for additional study related visits.

6.4 Withdrawal of Participants

Participants have the right to withdraw from the study at any time for any reason. It is understood by all concerned that an excessive rate of withdrawals can render the study un-interpretable; therefore, unnecessary withdrawal of patients should be avoided. Should a patient decide to withdraw from the study, all efforts will be made to report the reason for withdrawal as thoroughly as possible by completing the appropriate eCRF. Should a patient decide to withdraw from study, efforts will be made to explain the importance of remaining on trial follow up and seek permission to continue to allow routine follow-up data (height, weight, history of symptoms, clinical examination, bloods including FBC, LFTs, U & E, INR) to be used for trial purposes (with parent / legal guardian consent).

Patients who undergo liver transplant following IMP administration during the study period will not be withdrawn from the study but will continue to undergo monitoring and data collection as specified in the schedule of assessments (section 7).

6.5 Replacement of Subjects

Eligible patients who do not receive IMP infusion following screening, or who do not tolerate the procedure will be replaced for the purpose of maintaining trial numbers.

6.6 Expected Duration of Trial

The end of the trial will be defined as the last patient 10 year annual follow up post IMP infusion. Each individual subject will remain on the trial for 10 years post IMP infusion. Patients will be followed up intensively for the first 52 weeks as part of the main study (outlined in section 7.1). Patients will also be followed up long term for safety until 10 years post HMB002 infusion (which will be aligned with standard of care).

7. Trial Procedures

7.1 Schedule of Assessments

Study Visits [q]	Screening Days -3 to 0 [a]	Day 0	Day 1	Day 2	Day 3	Day 4	Day 5	Day 6	Day 7	Week 2	Week 4	Week 8	Week 12	Week 16	Week 24 (6 months)	Week 52 (12 months)	Unscheduled (within 52 wks)	Long term follow up (up to 10 years) [r]
Informed consent (parent/legal guardian)	X																	
Review Inclusion & exclusion	X	X																
Pre-infusion Checks [b]		X																
HMB002 Infusion		X																
Physical examination	X	X	X	X	X	X	X	X	X	X	X	X	X	X	X	X	X	
Medical and medication history	X																	
Urine or serum pregnancy test [c]	X																	
Height and weight	X									X	X	X	X	X	X	X		
Intra-abdominal Pressure [d]		X	x															
IMP infusion site review [e]		X	X															
Vital signs [f]	X	X	X	X	X	X	X	X	X	X	X	X	X	X	X	X	X	
Supportive Treatments [g]		(X)	(X)	(X)	(X)	(X)	(X)	(X)	(X)	(X)	(X)	(X)	(X)	(X)	(X)	(X)		
Clinical Bloods Tests 1 [h]	X	X [h]	X	X	X	X	X	X	X	X	X	X	X	X	X	X		X
Clinical Blood Tests 2 [i]	X	X [i]	X	X	X	X	X	X	X	X								
Bloods for translational research [j]	X	X [j]	X		X				X	X			X		X	X		
Neurological Assessments [k]	X	X	X	X	X	X	X	X	X	X	X	X	X	X	X	X		
Concomitant medications	X	X	X	X	X	X	X	X	X	X	X	X	X	X	X	X	X	X
Adverse events/SAEs	X	X	X	X	X	X	X	X	X	X	X	X	X	X	X	X	X	X
Quality of Life questionnaire [l]	X															X		
Ultrasound of the abdomen	X	X	X										X		X	X		X
Status Form		X	X	X	X	X	X	X	X	X	X	X	X	X	X	X		X
Microbeads Retrieval [m]																		
Discharge Post IMP Infusion [n]																		
Liver Transplant Form [o]																		
Withdrawal/ End of study [p]																		X

Table 3: Schedule of Assessments

[a] Screening and infusion may take place on the same day (Day 0) depending on clinical decision by delegated physician

[b] HMB002 infusion will be performed on Day 0 following complete review of all inclusion and exclusion criteria and completion of all pre-infusion checks. See section 4.3 Dosing regimen for details on dosing intervals between subsequent patients.

[c] Urine or serum pregnancy test (in cases of oliguria) will be performed in females of child bearing potential (FOCBP). FOCBP will only be included after a confirmed negative pregnancy test at screening. Pregnancy is an absolute contraindication to inclusion into the study. However, patients will be hospitalised at the time of screening and until recovery of their native liver or after transplant. Hence, during hospitalisation it is assumed that patients will be sexually abstinent, which is considered a highly effective method of contraception. Therefore, females of child bearing age are eligible for the trial without need for any additional testing or contraception. Should the patient survive with or without liver transplantation, participants will be advised as to highly effective methods of contraception if this is this is appropriate.

[d] Intra-abdominal pressure will be measured using urinary catheter where possible, at pre-dose (10 minutes prior to start of IMP infusion) and post dose: 1 hr, 8 hrs and 24 hrs (day1).

[e] IMP Infusion site to be reviewed at 2 hrs, 4hrs, 6 hrs, 8hrs,12 hrs and 24 hrs post infusion.

[f] Vital signs include Blood Pressure (BP), Pulse Rate (PR), Respiratory Rate (RR), Temperature and Oxygen Saturation (0_2 Sats). On Day 0, vital signs will be taken at Pre-dose (approximately 30 mins and 10 mins prior to start of IMP infusion) and Post-dose: 30mins, 1hr, 1.5hrs, 2 hrs, 2.5 hours, 3 hrs, 3.5 hrs 4 hrs, 8 hrs, 12 hrs and 24 hrs (Day1).

[g] Recording of ventilator settings, ionotropic support (including drugs and dose) and need for renal replacement therapy will be collected as part of standard supportive treatment. On Day 0, supportive treatment data will be collected at pre-dose (30 mins and 10 mins prior to start of IMP infusion and post dose: 30mins, 1hr, 1.5hr, 2hrs, 2.5hrs, 3hrs, 3.5hrs, 4hrs, 8hrs, 12 hrs & 24 hrs(Day 1).

[h] Clinical Blood Tests 1 include haematology (full blood count and differentials), clotting factors (INR, APTT, fibrinogen) liver function tests (ALT, AST, Creatine Kinase, Total bilirubin, Conjugated bilirubin (only done at screening and if clinically applicable), ALP, Albumin, total protein), urea and electrolytes (sodium, potassium, chloride, urea, creatinine) and Ammonia. At Day 0, bloods will be taken pre-dose (between -4 to -1 hour prior to IMP infusion) and post dose: 1 hr, 8hrs, 16hrs and 24hrs (day1).

[i] Clinical Blood tests 2 include blood glucose, lactate and blood gases (pH, Partial pressure of O2, Partial pressure of carbon dioxide, standard bicarbonate). On Day 0, these blood will be taken pre dose (10mins prior to start of IMP infusion) and post dose: 1hr, 2hrs, 4hrs, 8hrs, 16hrs, 24hrs (day1).

[j] Bloods for translational research on Day 0 – to be taken approximately 10 mins prior to IMP infusion and 1 hour post IMP infusion. Aliquots of certain human products (fresh frozen plasma, cryoprecipitate and albumin) administered as part of standard of care will also be sent as controls.

[k] Neurological Assessment includes Glasgow Coma Scale (GSC) and Pupil response.

[l] PedsQL™ Quality of Life Inventory questionnaires for parent and child will be optional; completed at screening and week 52 visits.

[m] The beads will be removed using laparoscopy prior to discharge of the patient from hospital or washed out at the time of transplantation, whichever occurs first. Usually this will be within 4 weeks of the procedure but in any case, microbeads will be retrieved within 24 weeks of HMB002 infusion.

[n] Liver Transplant form will be completed for those patients who go onto receive a transplant within the duration of the study.

[o] Patients will be discharged from hospital following recovery with either native liver or post liver transplant. Discharge post IMP infusion form to be completed.

[p] End of study/withdrawal form will be completed following completion of the study or for those patients who did not complete the study.

[q] All outpatient study visits following discharge, will have a flexible window of +/- 3 days except week 52 visits where +/- 7 days is permitted.

[r] Long term follow up data will be collected annually within +/- 1 month window. FBC, LFT and abdominal ultrasound will be collected annually until year 5 post IMP. SAEs and associated concomitant medications (except SAEs excluded from reporting) will be collected until end of study.

7.2 Study Assessments by the Visit

Informed Consent & Screening Assessments

The Investigator will provide trial information to parents/legal guardians of children who are considered to meet the study eligibility criteria. If appropriate children will also be given information about the trial. It is unlikely that they will be in a position to give assent due to encephalopathy/sedation for ventilation, but age-appropriate patient information sheets will be made available. This information should be sufficient to allow patients/parents/legal guardians to make an informed decision about participation.

Following informed consent from parent/legal guardian, the Investigator will conduct a full screening evaluation to ensure that the patient meets all inclusion and exclusion criteria (see section 6). All screening procedures will be carried out following consent, according to the study visit schedule. Certain routine assessments conducted as standard of care (e.g. height and weight) do not require informed consent and may be provided as screening data, if conducted within the permitted screening window prior to IMP infusion.

- Parent Information and Informed consent
- Medical and Medication History (received 6 months prior to consent)
- Physical examination
- Height and weight monitoring,
- Vital signs (body temperature BP, PR, RR, Oxygen saturation)
- Urine or serum pregnancy test in females of childbearing potential
- Clinical blood tests 1 and 2
- Bloods for translational research
- Collection of neurological parameters
- US of the abdomen
- Completion of PedsQL™ questionnaires
- Recording of Adverse events
- Recording of concomitant Medications

Pre-procedure monitoring, checks and IMP infusion

The procedure will be undertaken in the paediatric intensive care unit or High dependency Unit King's College Hospital, as children with acute liver failure generally require this degree of monitoring in any case. The child may be intubated and ventilated on clinical grounds. Haemofiltration and ionotropic support may be required according to the clinical condition of the patient and will not interfere with the procedure. Children may need blood products prior to the procedure in order to correct their INR to <2 (though this may not be achievable), platelet infusion should be given if platelet count is <50,000/ul and fibrinogen should be >1.0 g/l. Haemoglobin should be >7g/dL prior to the procedure. Where possible intra-abdominal pressure will be measured pre-infusion using urinary catheter. Pre-infusion Checks form will be completed prior to dosing with HMB002.

Pre-infusion checks

i. Correction of coagulopathy
ii. Haemoglobin > 7g/dl
iii. Patient has not got tense ascites
iv. Patient is sufficiently stable to tolerate procedure

v. Patient diagnosis / status has not changed from time of screening and consent
vi. No organ transplant is available.
vii. Full PICU / NICU / HDU monitoring in place and staffing sufficient to provide the same monitoring following the infusion.

A site will be selected under ultrasound guidance in the anterior abdominal wall to insert a cannula (14-16G). Local anaesthetic (1% lignocaine) can be used prior to insertion of the cannula. The position of the cannula can be checked with ultrasound, a connector attached and the cannula flushed with 5mL saline to make sure that it is in an appropriate position for infusion of beads. Insertion of the cannula will be done using aseptic technique. The solution containing beads will be infused manually into the peritoneal cavity with usually a 50ml sterile syringe, as a single or several infusions, to achieve in excess of 25 million hepatocytes per kilogram of the body weight. The volume of infusion will be determined by the patient's weight. The infusion will be administered at a rate of approximately 150-200ml per hour with close monitoring. The cannula could be left in place for up to one week. Anti-rejection drugs will not be used as alginate gel is expected to act as barrier against lymphocytes that mediate rejection. All patients should have prophylaxis with antibiotics as per King's College Hospital paediatric acute liver failure microbiology guidelines. The antibiotic choice will be influenced by patient characteristics (allergy, renal function, etc) and by previous exposure / known infectious agent susceptibility or colonisation.
Details regarding whether or not patient tolerated the full infusion and dose administered will be recorded on the Infusion details form. An infusion of more than 80% of the final product for the patient will be considered as a completed infusion.

Monitoring pre and post-infusion: Day 0 to Day 1:
Children will undergo continuous cardiorespiratory monitoring as per PICU/NICU/HDU standard of care for 24-hour post procedure. The patients will undergo at least daily examination, while still an inpatient. The following tests will be performed and data collected as outlined in the schedule of assessments, section 7.1.

- Physical Examination (Day 0)
- Intra-abdominal pressure will be measured using urinary catheter where possible, at pre-dose (10 minutes prior to start of IMP infusion) and post dose: 1 hr, 8 hrs and 24 hrs (day1).
- IMP infusion site review at 2 hrs, 4hrs, 6 hrs, 8hrs, 12 hrs and 24 hrs post infusion.
- Vital signs - Pre-dose (approximately 30 mins and 10 mins prior to start of IMP infusion) and Post-dose: 30mins, 1hr, 1.5hrs, 2 hrs, 2.5 hours, 3 hrs, 3.5 hrs 4 hrs, 8 hrs, 12 hrs and 24 hrs (Day1).
- Clinical Blood tests 1 pre-dose (-4 to -1 hour prior to IMP infusion) and post dose: 1 hr, 8hrs, 16hrs and 24hrs (day1).
- Clinical Blood tests 2 – pre-dose (within 10mins prior to start of IMP infusion) and post dose: 1hr, 2hrs, 4hrs, 8hrs, 16hrs, 24hrs (day1).
- Bloods for translation research taken pre-dose (approximately 10 mins prior to IMP infusion) and 1 hr and 24hrs (Day1) post infusion
- Collection of neurological parameters (Day 0 and Day 1)
- Recording of ventilator settings, inotropic support (list drugs and dose), need for renal replacement therapy (if required) at pre-dose (30 mins and 10 mins prior to start of IMP infusion) and post dose: 30mins, 1hr, 1.5hr, 2hrs, 2.5hrs, 3hrs, 3.5hrs, 4hrs, 8hrs, 12 hrs and 24 hour (Day 1).

- US of the abdomen (Day 0 and Day 1 post dose)
- Recording of Adverse events
- Recording of concomitant Medications
- Completion of the status form

Study Visits Day 2 to Day 7
All time points for the tests below are detailed in the schedule of assessments.

- Physical examination
- Vital signs (body temperature BP, PR, RR, Oxygen saturation)
- Clinical bloods tests 1 and 2
- Bloods for translation research
- Collection of neurological parameters
- Recording of ventilator settings, inotropic support (list drugs and dose), need for renal replacement therapy (if required)
- US of the abdomen
- Recording of Adverse events
- Recording of concomitant Medications
- Completion of the status Form

Microbead retrieval:
The beads will be removed using laparoscopy prior to discharge of the patient from hospital or at time of transplantation if this occurs. The timing of this is not possible to predict as it will depend on the time taken for the patient to recover to normal or near normal native liver function, or the time it takes to find a suitable organ for transplantation if the native liver does not recover. Usually this will be within 4 weeks of the procedure but in any case, microbeads should be retrieved within 24 weeks of HMB002 infusion.

The microbead retrieval form and discharge post IMP details will be recorded following the completion of these events during the study.

Study visit week 2:
- Physical examination
- Height and weight monitoring,
- Vital signs (body temperature BP, PR, RR, Oxygen saturation)
- Clinical blood tests 1 and 2
- Bloods for translational research
- Collection of neurological parameters
- Recording of ventilator settings, inotropic support (list drugs and dose), need for renal replacement therapy (if required)
- Recording of Adverse events
- Recording of concomitant Medications
- Completion of the status form

Study visit week 4:
- Physical examination
- Height and weight monitoring,
- Vital signs (body temperature BP, PR, RR, Oxygen saturation)

- Clinical blood tests 1
- Collection of neurological parameters
- Recording of ventilator settings, inotropic support (list drugs and dose), need for renal replacement therapy (if required)
- Recording of Adverse events
- Recording of concomitant Medications
- Completion of the status form

Study visit week 8:
- Physical examination
- Height and weight monitoring,
- Vital signs (body temperature BP, PR, RR, Oxygen saturation)
- Clinical blood tests 1
- Collection of neurological parameters
- Recording of ventilator settings, inotropic support (list drugs and dose), need for renal replacement therapy (if required)
- Recording of Adverse events
- Recording of concomitant Medications
- Completion of Status Form

Study visit week 12:
- Physical examination
- Height and weight monitoring,
- Vital signs (body temperature BP, PR, RR, Oxygen saturation)
- Clinical blood tests 1
- Bloods for translational research
- Collection of Neurological Parameters
- Recording of ventilator settings, inotropic support (list drugs and dose), need for renal replacement therapy (if required)
- Recording of Adverse events
- Recording of concomitant Medications
- US of the abdomen
- Completion of Status Form

Study visit week 16:
- Physical examination
- Height and weight monitoring
- Vital signs (body temperature BP, PR, RR, Oxygen saturation)
- Clinical blood tests 1
- Collection of neurological parameters
- Recording of ventilator settings, inotropic support (list drugs and dose), need for renal replacement therapy (if required)
- Recording of Adverse events
- Recording of concomitant Medications
- Completion of Status Form

Study visit week 24:
- Physical examination

- Height and weight monitoring,
- Vital signs (body temperature BP, PR, RR, Oxygen saturation)
- Clinical blood tests 1
- Bloods for translational research
- Collection of neurological parameters
- Recording of ventilator settings, inotropic support (list drugs and dose), need for renal replacement therapy (if required)
- Recording of Adverse events
- Recording of concomitant Medications
- US of the abdomen
- Completion of Status Form

Study visit week 52:

- Physical examination
- Height and weight monitoring,
- Vital signs (body temperature BP, PR, RR, Oxygen saturation)
- Clinical blood tests 1
- Bloods for translational research
- Collection of neurological parameters
- Recording of ventilator settings, inotropic support (list drugs and dose), need for renal replacement therapy (if required)
- Recording of Adverse events
- Recording of concomitant Medications
- Completion of PedsQL™ questionnaires
- US of the abdomen
- Completion of Status Form

Study Follow up

Participants will be seen in clinic as per the study visit schedule for one year post IMP dosing.

Long Term Safety Follow Up

Safety monitoring will be conducted until 10 years after HMB002 infusion. This will be aligned with routine care. Routine follow up will be conducted annually as a minimum but may be more frequent should the clinical condition of the child or young person require this. For example, if the child has undergone liver transplantation, follow up will be frequent and lifelong.

FBC, LFTs and abdominal ultrasound data will be collected annually during the long term follow up period (years 2 to 5). SAEs will be collected from week 52 until 10 years post IMP infusion, however certain expected SAEs (detailed in section 9.2) which are known complications in liver transplant patients will be excluded from reporting to sponsor. After the 2 year post IMP safety data is completed, reporting of SAEs will be reviewed with the DSMB and an amendment may be submitted to rationalise the SAEs reported to sponsor/MHRA.

7.3 Laboratory Tests

Clinical Blood Tests

For the purposes of the study the pre- and post-infusion blood tests (clotting, haematology, biochemistry including ammonia, lactate, venous blood gas) will be collected at times specified in the schedule of assessments section 7.1 and 7.2.

All clinical blood tests will be taken as per routine care (Full blood count, Urea and Electrolytes, Liver Function Tests, International Normalised Ratio, ammonia, lactate) in the appropriate containers and analysed by Viapath, King's College Hospital with results available on the electronic patient record. A total volume of 7 – 10 mL blood will be taken at visits requiring clinical blood tests 1 and 2 together, and 5ml of blood for clinical blood tests 1 only (see schedule of assessments section 7.1).

Translational Research Blood Tests

Additional 1ml of blood will be taken for research assays at the same time as clinical blood tests. Consent for research blood samples will be optional. Plasma samples will be stored in the Liver Labs (Hepatocyte Lab), Institute of Liver Studies, 3rd Floor, Cheyne Wing, King's College Hospital, at -80°C. Further details provided in the HELP study laboratory manual.

Plasma samples will be shipped to the Translational Mass Spectrometry Research Laboratory, Institute of Child Health, UCL for HepaMorph panel and Inflammasome tests (proprietary tests in validation).

Bloods for translational research will be collected at times specified in the schedule of assessments section 7.1 and 7.2. Any surplus plasma sample will be transferred to King's College Hospital Paediatric Liver Biobank for long term storage (HTA License no: 12378), following consent from parent/legal guardian.

All research bloods and plasma samples will be labelled with the study participant identification number (PIN) and initials. Only the local research team will have access to the link between personal identifiable data and PIN number for enrolled participants.

Aliquots of certain human products (fresh frozen plasma, cryoprecipitate and albumin given to patients as part of standard of care, will be drawn from the product bag at the end of infusion of that product. These samples will also be labelled, frozen and sent together with patient samples for protein comparison and analysis.

8. Assessment of Efficacy

8.1 Primary Efficacy Parameters

Efficacy is demonstrated by survival of the patient with native liver.

8.2 Secondary Efficacy Parameters

Parameters indicating native liver function such as neurological parameters, need for intensive care support, blood products, blood tests including ammonia, lactate, INR, AST, ALT,

bilirubin and albumin. (See clinical blood tests 1 and 2 in schedule of assessments). PedsQL™ questionnaires will also be administered at baseline (screening) and at 12 months post infusion.

8.3 Procedures for Assessing Efficacy Parameters

Primary efficacy is survival and is confirmed at clinic visit.

Secondary efficacy parameters are determined using monitoring (electronic monitoring while an inpatient and manual BP / PR / RR and oxygen saturation. Blood parameters: venepuncture 7 – 10 mL for clinical blood tests 1 and 2 together and 5ml of blood for clinical blood tests 1 only (see schedule of assessments).
Quality of life will be assessed at screening and 12 months using an internationally validated PedsQL™ questionnaires for transplant recipients.

9. Assessment of Safety

9.1 Specification, Timing and Recording of Safety Parameters.

Safety parameters and adverse event (AE, SAE, SAR and SUSAR) information will be collected from the point of parent/legal guardian consent until week 52 post IMP administration (as outlined in section 7).

During the infusion and for at least 24 hours afterwards the patient will be continuously monitored in PICU / NICU / HDU. Intra-abdominal pressure will be monitored via the bladder if possible. Safety will be assessed using history of symptoms which have occurred or worsened since study commencement, physical examination, blood tests including white cell count, haemoglobin, platelets, Urea and electrolytes, liver function tests, lactate, venous blood gas.

While an inpatient, need for intervention such as escalation of care will also be recorded. Ultrasound may be used to assess for intra-abdominal bleeding.

Long term follow up is important for phase 1 first in human ATIMP trial. During long term follow up (post week 52 visit), AEs and SAES will be documented in the medical notes as per routine care practise and reviewed by clinicians. However, only SAEs, SARs and SUSARS will be reported (excluding known complications following liver transplant outlined in section 9.2).

9.2 Procedures for Recording and Reporting Adverse Events

Reporting Definitions

The Medicines for Human Use (Clinical Trials) Regulations 2004 and Amended Regulations 2006 gives the following definitions:

- **Adverse Event (AE):** Any untoward medical occurrence in a subject to whom a medicinal product has been administered including occurrences which are not necessarily caused by or related to that product.

- **Adverse Reaction (AR):** Any untoward and unintended response in a subject to an investigational medicinal product which is related to any dose administered to that subject.
- **Unexpected Adverse Reaction (UAR):** An adverse reaction the nature and severity of which is not consistent with the information about the medicinal product in question set out in the Investigator's Brochure (IB) relating to the trial in question.

Serious adverse Event (SAE), Serious Adverse Reaction (SAR) or Suspected Unexpected Serious Adverse Reaction (SUSAR): Any adverse event, adverse reaction or unexpected adverse reaction, respectively, that

- Results in death;
- Is life-threatening;
- Required hospitalisation or prolongation of existing hospitalisation;
- Results in persistent or significant disability or incapacity;
- Consists of a congenital anomaly or birth defect.

Important Medical Events (IME) & Pregnancy

Events that may not be immediately life-threatening or result in death or hospitalisation but may jeopardise the patient or may require intervention to prevent one of the other outcomes listed in the definition above should also be considered serious.

Although not a serious adverse event, any unplanned pregnancy will also be reported via the SAE reporting system.

Assessment of Adverse Events

- Documentation of the AEs in the eCRF will be according to the following criteria:
- Description of the AE: diagnosis if known, with signs and symptoms, giving details appropriate to the event,
- Dates of onset and resolution of the AE,
- Severity, grading –using the NCI Common Terminology Criteria for Adverse Events (CTCAE, version 4.0) and detailed below. If not included in CTCAE, this will be a clinical decision and graded as detailed below in severity of adverse events section.
- Assessment of causal relationship to study treatment (see below)
- Action taken regarding study treatment: none / infusion discontinued / infusion delayed
- Outcome: complete recovery/not yet recovered/recovered with sequelae/death/unknown. The investigator may be asked to provide follow-up information and/or discharge summaries as needed.

Assessment of Causal Relationship to Study Product

The assignment of causality should be made by the investigator responsible for the care of the participant and discussed with the Chief Investigator (CI) in cases where causality is doubtful.

Category	Definition
Definitely:	There is clear evidence to suggest a causal relationship, and other possible contributing factors can be ruled out.
Probably:	There is evidence to suggest a causal relationship, and the influence of other factors is unlikely
Possibly	There is some evidence to suggest a causal relationship (e.g. the event occurred within a reasonable time after administration of the trial medication). However, the influence of other factors may have contributed to the event (e.g. the participant's clinical condition, other concomitant events).
Unlikely	There is little evidence to suggest there is a causal relationship (e.g. the event did not occur within a reasonable time after administration of the trial medication). There is another reasonable explanation for the event (e.g. the participant's clinical condition, other concomitant treatments).
Unrelated	There is no evidence of any causal relationship.

Severity of Adverse Events

- The severity assessment, described as the clinical intensity determination, for an AE/SAE should be completed using the NCI Common Terminology Criteria for Adverse Events (CTCAE, version 4.0).
- Any AE/SAE not specified in the CTCAE version 4.0 will be graded as follows:
  Mild: asymptomatic or mild symptoms – clinical or diagnostic observations only
  Moderate: minimal, local or non-invasive intervention indicated
  Severe: medically significant but not immediately life-threatening, hospitalisation indicated
  Life-threatening consequences: urgent intervention needed
  Death related to AE

Safety Reporting Period

AEs and SAEs will be reported from the time of consent to Week 52 study visit. Any serious adverse events that are deemed related to the IMP (serious adverse reactions, SARs) will be considered unexpected (SUSARs), as outlined in the IB.

All liver transplants will be reported as an SAE. However, known complications below which are universal in patients post liver transplant will not be reported as SAEs to the sponsor, unless they result in death, are considered to be related to the study drug or worse than what would normally be expected.

These include organ rejection, infection or lymphoma related to immunosuppression, technical complications of liver transplantation (biliary, portal vein, hepatic artery and hepatic vein), incisional hernias related to transplant surgery and other complications directly related to immunosuppressive drugs.

During long term follow up (post week 52 visit), AEs and SAES will be documented in the medical notes as per routine care and reviewed by clinicians. However, only SAEs, SARs and SUSARS will be reported (excluding known complications following liver transplant as highlighted in the previous section).

Reporting Responsibilities
King's College Hospital has delegated the delivery of the Sponsor's responsibility for Pharmacovigilance (as defined in Regulation 5 of the Medicines for Human Use (Clinical Trials) Regulations 2004 to the King's Health Partners Clinical Trials Office (KHP-CTO).

All SAEs, SARs and SUSARs will be reported immediately by the Chief Investigator or PI (and certainly no later than 24hrs) to the KHP-CTO in accordance with the current Pharmacovigilance Policy.

The KHP-CTO will report SUSARs to the regulatory authorities (MHRA, competent authorities of other EEA (European Economic Area) states in which the trial is taking place.
The Chief Investigator will report to the relevant ethics committee. Reporting timelines are as follows:

- SUSARs which are fatal or life-threatening must be reported not later than 7 days after the sponsor is first aware of the reaction. Any additional relevant information must be reported within a further 8 days.
- SUSARs that are not fatal or life-threatening must be reported within 15 days of the sponsor first becoming aware of the reaction.
- The Chief Investigator and KHP-CTO (on behalf of the sponsor), will submit a Development Safety Update Report (DSUR) relating to this trial IMP, to the MHRA and REC annually.

The CI will submit annually to the main REC an Annual Progress Report.
All SAEs, SARs and SUSARs (including any follow-up information), will be reported using the KHP-CTO SAE report form.

Pregnancy
Should a trial participant become pregnant during the trial, she will be followed up for safety until the birth of the child. Although not a serious event, any unplanned pregnancy should be reported via the SAE reporting system.

9.3 Treatment Stopping Rules

Premature discontinuation of study
The trial may be prematurely discontinued by the Sponsor, Chief Investigator or Regulatory Authority on the basis of new safety information or for other reasons given by the DSMB, regulatory authority or ethics committee concerned. The Sponsor and CI reserve the right to stop the trial at any time, for any justifiable reason.

In the event of premature termination, the Sponsor will notify the regulatory authorities within 15 days by providing a detailed written explanation. The CI will inform the REC. The

affected trial participants will also be informed promptly and appropriate follow-up visits will be arranged. No further participant data will be collected.

The clinical trial may be prematurely terminated for the following reasons:

- Serious and/or persistent non-compliance with trial protocol
- Non-compliance with ethical standards, regulatory requirements or GCP compliance
- Findings uncovered during monitoring visits, audits or inspections that compromise patient safety or suitability of the site to act as a trial centre
- Recommendation from DSMB
- Failure to meet recruitment targets

During the course of the study, any of the following events will trigger a halt in patient recruitment and a safety meeting of the DSMB:

- Death or life-threatening event occurs due to a reaction to the product
- Or 2 or more ATIMP related SAEs of non-lethal non-life threatening reactions to HMB002.

The trial will be put on hold pending a safety investigation in either case above. If following an internal safety review the Sponsor deems it appropriate to restart the trial, this can be done after approval of a substantial amendment.

10. Statistics

The trial is based on a two-stage design. The initial decision of whether the trial proceeds will be based on the response of the first 9 patients recruited into the study (stage 1). If two or fewer survive with the native liver at 24 weeks then the trial will be stopped at this stage. If more than 2 survive with the native liver, then recruitment will continue to a total of 17 patients (stage 2).

If the trial continues then the proportion of individuals who survive with the native liver to 24 weeks will be estimated with a 95% confidence interval. Survival with the native liver at 52 weeks will also be estimated.

10.1 Sample Size

A Simon's two-stage design for a one-sample exact test will be estimated, assuming a one-year survival rate of 0.20 or less under the null hypothesis, and 0.5 or more under the alternative, with 80% power and 5% type I error rate. 9 patients will be recruited into the study in the first stage; this will be extended to 17 patients as part of the Simon two stage design if there is evidence to support continuation (see section 6.3.1).

10.2 Analysis

All patients enrolled will be followed up and included in the final analysis unless they do not receive HMB002 infusion.

Patients will be described in terms of their baseline characteristics using frequencies and percentages, mean and standard deviation or median and range as appropriate.

The proportion of individuals who survive with their native liver at 24 weeks will be presented with a 95% confidence interval.

The proportion who survive with their native liver at 52 weeks and the proportion who survive at 24 and 52 weeks will also be presented with 95% confidence intervals.

Adverse and serious adverse events will be described.

A detailed plan will be given in a statistical analysis plan.

Interim Analysis
An interim analysis will be conducted at stage 1 Go/No GO time point for DSMB safety review, when the 9th patient has completed their 24-week post IMP study follow up.

One year Follow up Analysis
This will be conducted following database lock after the 52-week post IMP follow up data has been completed and monitored for all patients included in stage 1 & 2. Alternatively, if the trial does not progress to stage 2, database will be locked following 52-week post IMP follow up is completed for all stage 1 patients.

11. Study Management

11.1 Trial Management Group (TMG)

This TMG will be formed comprising the CI, other lead investigators, core study team including statisticians, clinical trial manager, chief scientist research, research nurse, GMP team. The trial management team will be responsible for the day to day management of the trial activities and will meet on a regular basis to discuss any trial related activities or issues.

11.2 Data and Safety Management Board (DSMB)

In view of the need for rapid decision making and a high level of involvement by board members who will need to be experts in the field of paediatric liver failure and first in man studies, the study will be overseen by a single data and safety monitoring board (DSMB). The DSMB will be constituted prior to study opening, comprising of an Independent Chair, Independent Clinicians (s) and an Independent Statistician. The DSMB will review individual and cumulative data to evaluate safety, study conduct, scientific validity and integrity of the trial. The DSMB will meet prior to initiation of study recruitment to agree on the type and format of data reports and sign the DSMB charter.
Further details on the DSMB membership and terms of reference will be provided in the DSMB charter.
Timing of Meetings subject to agreement by members of DSMB are:

- prior to initiation of study recruitment
- Safety data review 4 weeks after IMP infusion of first patient
- Safety data review 4 weeks after IMP infusion of fourth patient
- Safety data review 24 weeks after IMP infusion of 9th patient (End of stage 1 - GO/ No GO Decision)

- Safety data review at 52 weeks after IMP infusion of 17th patient
- Safety data review at 2 years after IMP infusion of all patients and subsequent review during the long term follow up period.

DSMB meetings will also be convened if:

- Death or life-threatening event occurs due to a reaction to the product
- 2 or more serious cases of non-lethal non-life-threatening reactions to HMB002.
- Or at any time deemed necessary by the DSMB Chair due to safety concerns arising at any time during the duration of the study.

12. Ethics & Regulatory Approvals

12.1 Good Clinical Practice

The trial will be conducted in compliance with the principles of the Declaration of Helsinki (1996), the principles of GCP and in accordance with all applicable regulatory requirements including but not limited to the Research Governance Framework and the Medicines for Human Use (Clinical Trial) Regulations 2004, as amended in 2006 and any subsequent amendments.

12.2 Ethics Committee Approval

This protocol and related documents will be submitted for review to West London & GTAC Research Ethics Committee (REC), and to the Medicines and Healthcare products Regulatory Agency (MHRA) for Clinical Trial Authorisation.

The Chief Investigator will submit a final report at conclusion of the trial to the KHP-CTO (on behalf of the Sponsor), the REC and the MHRA within the timelines defined in the Regulations.

12.3 Informed Consent Procedure

It is the responsibility of the Chief Investigator, or person to whom the Investigator delegates the responsibility, to obtain written informed consent for each parent/legal guardian prior to performing any trial related procedure in compliance with regulations. All trial investigators seeking consent must have received Human Tissue Act training for the taking of consent involving tissues and cells used as part of the trial, be up-to-date with their GCP training and delegated for the task on the Delegation Log.

Investigators must ensure that they adequately explain the parent information leaflet (PIL) outlining the aim, trial treatment, potential risks and benefits of taking part in the trial. The patient's parent/legal guardian should be given ample time to read the PIL and to discuss their child's participation with others outside of the clinical research team. However, due to the acute nature of their condition, the available time may be less than 24 hours. The parent / legal guardian must be given an opportunity to ask questions which should be answered to their satisfaction. The right of the parent/guardian to refuse to participate without giving a reason must be respected.

The trial includes both children and young adults < 16 years and written assent will be obtained from the patient whenever it is possible to do so (as appropriate to age and legislation). If capable, and under appropriate circumstances, minors should be approached to provide assent by a delegated clinician. Age-and-state-of-development IEC-approved Patient Information Sheet and Assent forms, describing (in simplified terms) the details of the study intervention/product, study procedures and risks should be used. The minor should personally write their name (or initial) and date the assent form, which is then signed by the delegated clinician taking consent. Assent forms do not substitute for the consent form signed by the patient's legally acceptable representative. Assent should be taken where appropriate and documented in the patient notes, however the absence of assent does not exclude the patient, provided consent has been obtained from the parent/legal guardian. Though assent will be sought from children themselves however, given that they will have acute liver failure they are unlikely to be able to give informed assent (they may be sedated and ventilated or encephalopathic as per inclusion criteria).

If the parent/legal guardian decides for their child to participate in the trial they must be asked to sign and date the latest approved version of the Informed Consent Form (ICF). The form must also be signed and dated by the PI or delegate involved in the informed consent process. Details of the informed consent and assent discussions should be recorded in the patient's medical notes. This should include date and content of the initial discussion, the date consent was obtained and trial name.

A copy of the PIS and signed consent form and/or assent forms will be given to the patient / parent / guardian and a copy will be kept in their medical records. The original signed consent/assent forms will be kept in the Investigator Site File.

13. Quality Assurance

Monitoring of this trial will be to ensure compliance with Good Clinical Practice and scientific integrity will be managed and oversight retained, by the KHP-CTO Quality Team.

13.1 Trial Monitoring

The KHP-CTO Clinical Research Associate (CRA) will be responsible for monitoring essential documents at site and perform source data verification (SDV). The trial will be monitored by the KHP-CTO CRA on behalf of the co-sponsors according to the trial risk assessment and the monitoring plan established during study start-up.

13.2 Data Handling

The CI will act as custodian for the trial data. The following guidelines will be strictly adhered to:

- Patient data will be pseudo-anonymised
- All trial data will be stored in line with the Medicines for Humans Use (Clinical Trials) Amended regulations 2006, the Data protection Act 2018 and GDPR (and all amendments to follow).

- All trial data will be archived in line with the Medicines for Humans Use (Clinical Trials) Amended regulations 2006 and as defined in the King's Health Partners Clinical Trials Office Archiving SOP (and all amendments to follow).

13.3 Direct Access to Source Data and Documents

The CI/PI must allow the Sponsor, designated trial monitors, and when necessary, members of the REC or representatives of the regulatory authorities to review, monitor, audit and/ or inspect the trial by providing direct access to source data and other documents (e.g. patients' case sheets, blood test reports, histology reports etc.). During such activities, the confidentiality of personal data will be respected at all times. By signing the ICF, the recipient will specifically consent to direct access to his/her medical records and source documentation for the purpose of SDV and regulatory inspection.

14. Data Management

A specific data management plan will be created for the study.

14.1 Data Collection

A web based electronic data capture (EDC) system will be designed, using the InferMed Macro 4 system. This system is fully validated and regulatory compliant (GCP, 21CRF11, EC Clinical Trial Directive). The EDC will be created in collaboration with the trial statisticians and the CI and maintained by the King's Clinical Trials Unit. It will be hosted on a dedicated secure server within KCL.

No identifiable data beyond participant initials and date of birth will be entered on the EDC or transferred to the KCTU. No data will be entered onto the EDC system unless a participant's parent/legal guardian has signed a consent form. Source data will be entered by authorised site staff onto the EDC by going to www.ctu.co.uk and clicking the link to access the MACRO 4 EDC system. A full audit trial of data entry and any subsequent changes to entered data will be automatically date and time stamped, alongside information about the user making the entry/changes within the system. Over the course of the trial, the Trial monitor will conduct on-site monitoring as outlined in the Trial Monitoring Plan. Where there are data queries raised the delegated site staff will be responsible for resolving the queries. The CI team will also undertake appropriate reviews of the entered data for the purpose of data cleaning and will request amendments as required.
Following completion of monitoring and data cleaning of all week 52 post IMP visit data, the site PI will review all the data for each participant and provide electronic sign-off to verify that all the data are complete and correct. At this point, all data can be formally locked for analysis.

Upon request, KCTU will provide a copy of the final exported dataset to the CI and delegated staff in .csv format and the CI will onward distribute as appropriate.

Safety monitoring data collected during the long term follow up period (week 52 to 10 years post IMP) will be recorded on a separate database and locked following PI review and sign off. A clinical study report will be submitted within 12 months of end of trial notification.

14.2 Source Data

Source data are defined as all the information in original records (and certified copies of original records) of clinical findings, observations, or other activities that are necessary for the complete reconstitution and evaluation of the trial.
Source documentation for the study includes, but is not limited to:

- Informed consent forms
- Medical records/clinical reports/laboratory reports/hospital correspondence/QoL questionnaires

The data entered into an eCRF should be verifiable with original source records kept at study centre.

14.3 Electronic CRF Database Access

The CI or delegate will request usernames and passwords from the KCTU. Database access will be strictly restricted through user-specific passwords to the authorised research team members. It is a legal requirement that passwords to the EDC are not shared, and that only those authorised to access the system are allowed to do so. If new staff members join the study, a user-specific username and password must be requested via the CI or delegate (e.g. Trial Manger) from the KCTU team and a request for access to be revoked must be requested when staff members leave the project. Study site staff experiencing issues with system access or functionality should contact the CI or delegate (e.g Trial Manger) in the first instance. Staff will receive training on the EDC system.

14.4 Archiving

At the end of the trial, all Essential Documentation will be archived for a minimum of 30 years in a GCP compliant archive facility. The documents that relate to ATIMP traceability will be retained for a minimum of 30 years and beyond the expiry date of the product. The TMF will be archived as per current KHP CTO SOPs. To enable peer review and/or audits from health authorities, all essential source and study documentation will be securely archived after study completion, in accordance with current regulatory requirements. Essential documents should be archived in a way that ensures that they are readily available, upon request, to the concerned authorities.

14.5 Publication Policy

All data and results generated from this trial are confidential. Agreement from the sponsors will be required prior to the disclosure of any trial related data.
It is intended that the results of the study will be reported and disseminated at international conferences and in peer-reviewed scientific journals. The chief investigator will ensure that the final results are analysed, transcribed, reported and disseminated at the end of the study.

This trial is subject to an external communications strategy which makes patients and healthcare providers aware of the study and to encourage recruitment.

15. Insurance / Indemnity

As the sponsor, King's College Hospital NHS Foundation Trust have clinical negligence cover as part of the NHS Clinical Negligence Scheme for Trusts.

16. Financial Aspects

Funding to conduct the trial is provided by the Medical Research Council DPFS grant, MRC Reference: MR/V038583/1.

Patients and their parent/legal guardians will be reimbursed for travel to and from King's College Hospital.

17. References

1. Bhaduri B and Mieli-Vergani G. Fulminant Hepatic Failure: Pediatric Aspects. Semin Liver Dis. 1996 Mar 17;16(04):349–55. Available from: https://doi.org/10.1055%2Fs-2007-1007248
2. Dhawan A. Acute liver failure in children and adolescents. Clin Res Hepatol Gastroenterol. 2012 Jun;36(3):278–83. Available from: https://doi.org/10.1016%2Fj.clinre.2012.03.022
3. Faraj W, Dar F, Bartlett A, Melendez HV, Marangoni G, Mukherji D, Vergani GM, Dhawan A, Heaton N and Rela M. Auxiliary Liver Transplantation for Acute Liver Failure in Children. Ann Surg [Internet]. 2010 Feb;251(2):351–6. Available from: https://doi.org/10.1097%2Fsla.0b013e3181bdfef6
4. Dhawan A, Puppi J, Hughes RD and Mitry RR. Human hepatocyte transplantation: current experience and future challenges. Nat Rev Gastroenterol Hepatol. 2010 May;7(5):288–98. Available from: www.nature.com/nrgastro
5. Dhawan A, Chaijitraruch N, Fitzpatrick E, Bansal S, Filippi C, Lehec SC, Heaton ND, Kane P, Verma A, Hughes RD and Mitry RR. Alginate microencapsulated human hepatocytes for the treatment of acute liver failure in children. J Hepatol. 2020;
6. Fitzpatrick E, Wu Y, Dhadda P, Hughes RD, Mitry RR, Qin H, Lehec SC, Heaton ND and Dhawan A. Co-culture with mesenchymal stem cells results in improved viability and function of human hepatocytes. Cell Transplant. 2013 Oct 18;24(1):73–83. Available from: http://www.ncbi.nlm.nih.gov/pubmed/24143888
7. Van Poll D, Parekkadan B, Cho CH, Berthiaume F, Nahmias Y, Tilles AW and Yarmush ML. Mesenchymal stem cell-derived molecules directly modulate hepatocellular death and regeneration in vitro and in vivo. Hepatology. 2008 May;47(5):1634–43. Available from: http://www.ncbi.nlm.nih.gov/pubmed/18395843
8. Lai RC, Arslan F, Lee MM, Sze NSK, Choo A, Chen TS, Salto-Tellez M, Timmers L, Lee CN, El Oakley RM, Pasterkamp G, de Kleijn DPV and Lim SK. Exosome secreted by MSC reduces myocardial ischemia/reperfusion injury. Stem Cell Res. 2010 May;4(3):214–22. Available from: https://doi.org/10.1016%2Fj.scr.2009.12.003
9. Xin H, Li Y, Buller B, Katakowski M, Zhang Y, Wang X, Shang X, Zhang ZG and Chopp M. Exosome-Mediated Transfer of miR-133b from Multipotent Mesenchymal Stromal Cells to Neural Cells Contributes to Neurite Outgrowth. Stem Cells. 2012 Jul;30(7):1556–64. Available from: https://doi.org/10.1002%2Fstem.1129
10. Jitraruch S, Dhawan A, Hughes RD, Filippi C, Soong D, Philippeos C, Lehec SC, Heaton ND, Longhi MS and Mitry RR. Alginate microencapsulated hepatocytes optimised for transplantation in acute liver failure. PLoS One. 2014;9(12):e113609.

18. Appendices

18.1 Appendix 1 – Acute Liver Failure Classification

Categories of ALF may be classified as the following:

1. Paracetamol toxicity (i) pH<7.25 more than 24 hours post dose and after adequate fluid resuscitation or (ii) INR> 6.5 and creatinine > 300 / anuria and grade 3- 4 encephalopathy or (iii) significant liver injury and coagulopathy following exclusion of other causes of hyperlactatemia arterial lactate > 5 on admission and > 4 subsequently in the presence of hepatic encephalopathy or (iv) 2 of 3 from INR> 6.5 / Cr > 300 umol/mL/ oliguria or grade 3 – 4 HE with clinical evidence deterioration (increasing FiO2, increasing inotropic requirements).
2. Favourable non-paracetamol aetiology (e.g. acute viral hepatitis): (i) hepatic encephalopathy plus INR> 6.5 or (ii) 2 of the following INR>3.5, any grade encephalopathy > 7 days from onset jaundice, bilirubin >300.
3. Non-favourable non-paracetamol aetiology (seronegative or drug induced): (i) INR >6.5 or in the absence of encephalopathy or (ii) INR> 2 (after vitamin K) and one of INR> 3.5 or time from jaundice to encephalopathy > 7 days or bilirubin > 300.
4. Acute presentation Wilson disease or Budd Chiari – coagulopathy and any encephalopathy.
5. ALF < 2 years INR > 4 or grade 3 – 4 encephalopathy.
6. ALF with at least 2 of the following. INR >4, Bilirubin >235umol/l, age < 2 years and White cell count >9 x 10^9/l.

* Children who meet inclusion criteria as above but would otherwise not be suitable for liver transplant because of progressive neurological disease for example, will not be excluded from the trial unless they also have exclusions as detailed in criteria for the trial.
